# Electrocatalytic C─N Coupling in Aqueous Solutions for High‐Value Product Synthesis

**DOI:** 10.1002/anie.202518370

**Published:** 2026-02-17

**Authors:** Yunpeng Zuo, Dongxue Yu, Xiaoran Zhang, Xin Wang

**Affiliations:** ^1^ Department of Chemistry City University of Hong Kong Hong Kong P.R. China

**Keywords:** aqueous solution, electrocatalytic C─N Coupling, high value‐added products

## Abstract

Organic nitrogen‐containing compounds, such as urea, amines, amides, and amino acids, play a crucial role in global biogeochemical cycles and industrial systems. Electrocatalytic C─N coupling, enabling reactions under mild conditions (room temperature/pressure) and utilizing a range of feedstocks (CO_2_, CO, N_2_, NO_3_^−^, and NH_3_, etc.), has emerged as a sustainable alternative for the synthesis of these chemicals. Recent advancements in diversifying product scopes via C─N coupling methodologies have significantly enhanced reaction versatility and conversion efficiency. These developments enable novel reaction pathways while addressing critical challenges, such as slow kinetics (e.g., high bond dissociation energies in N≡N or C═O bonds) and competing reactions. This review comprehensively examines the progress in electrochemical C─N coupling, with a focus on reaction mechanisms across diverse systems and the advanced catalysts driving these transformations. Furthermore, we summarize the integrated electrolytic cell design to bridge laboratory‐scale breakthroughs with sustainable large‐scale production. By overcoming existing limitations, electrocatalytic synthesis holds the potential to transform chemical manufacturing while mitigating environmental impacts.

## Graphical abstract

Electrocatalytic C–N coupling offers a sustainable route to synthesize crucial organic nitrogen compounds from abundant feedstocks under mild conditions. This review examines recent progress in overcoming key challenges, like slow kinetics and competing reactions through advanced catalysts and novel mechanisms, paving the way for transforming chemical manufacturing while reducing environmental impact.

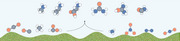

## 1 Introduction

Organic nitrogen‐containing compounds, formed through the coupling of carbon (C) and nitrogen (N), are essential components of the biomatrix [1, 2]. The C and N cycles form the core foundation of human civilization's development. Historically, the synthesis of C─N compounds has been a focal point of exploration [3, 4]. The earliest large‐scale industrial synthesis can be traced back to the Haber‐Bosch process, which operates under harsh conditions (100 bar, 400–500 °C). This process produces ammonia, which is subsequently used in thermal catalysis to synthesize various high‐value‐added organic C─N compounds [5, 6]. For instance, amines are synthesized through the amination of alcohols with NH_3_ at 100–250 °C and 50–250 bar, while industrial urea production relies on the Bosch‐Meiser process, which couples NH_3_ and CO_2_ at 200 °C and 210 bar [5, 6]. However, the Haber‐Bosch process (H‐B) relies heavily on hydrogen (H_2_) derived from methane gas reforming, a process that generates CO_2_ as a byproduct. As the typical energy‐intensive industrial process, H‐B consumes approximately 2% of the world's energy and contribute significantly to global CO_2_ and NO_x_ emissions [7, 8, 9, 10]. Furthermore, modern societal activities, transportation, agriculture, and petrochemical production have disrupted natural C and N cycles, released vast amounts of greenhouse gases and posed severe environmental and health challenges. In response to the global consensus on sustainable development, the scientific community has been exploring renewable energy‐driven green technologies to replace traditional energy‐intensive ones. One promising approach is the use of green hydrogen produced via water electrolysis to replace gray hydrogen from steam methane reforming (SMR) [11, 12, 13]. Another solution is the development of sustainable alternatives to the Haber‐Bosch process [14, 15, 16]. Electrocatalytic technology, with its advantages of energy efficiency, environmental friendliness, and mild reaction conditions, has emerged as a promising green solution for synthesizing C─N coupling products [17, 18, 19, 20]. This has sparked significant interest, particularly in heterogeneous electrosynthesis, which integrates electrochemistry and organic synthetic chemistry [21, 22, 23, 24]. Recent advancements have led to the development of novel catalysts and electrosynthesis designs, enabling the synthesis of diverse C─N compounds [25, 26, 27, 28, 29].

**FIGURE 1:**
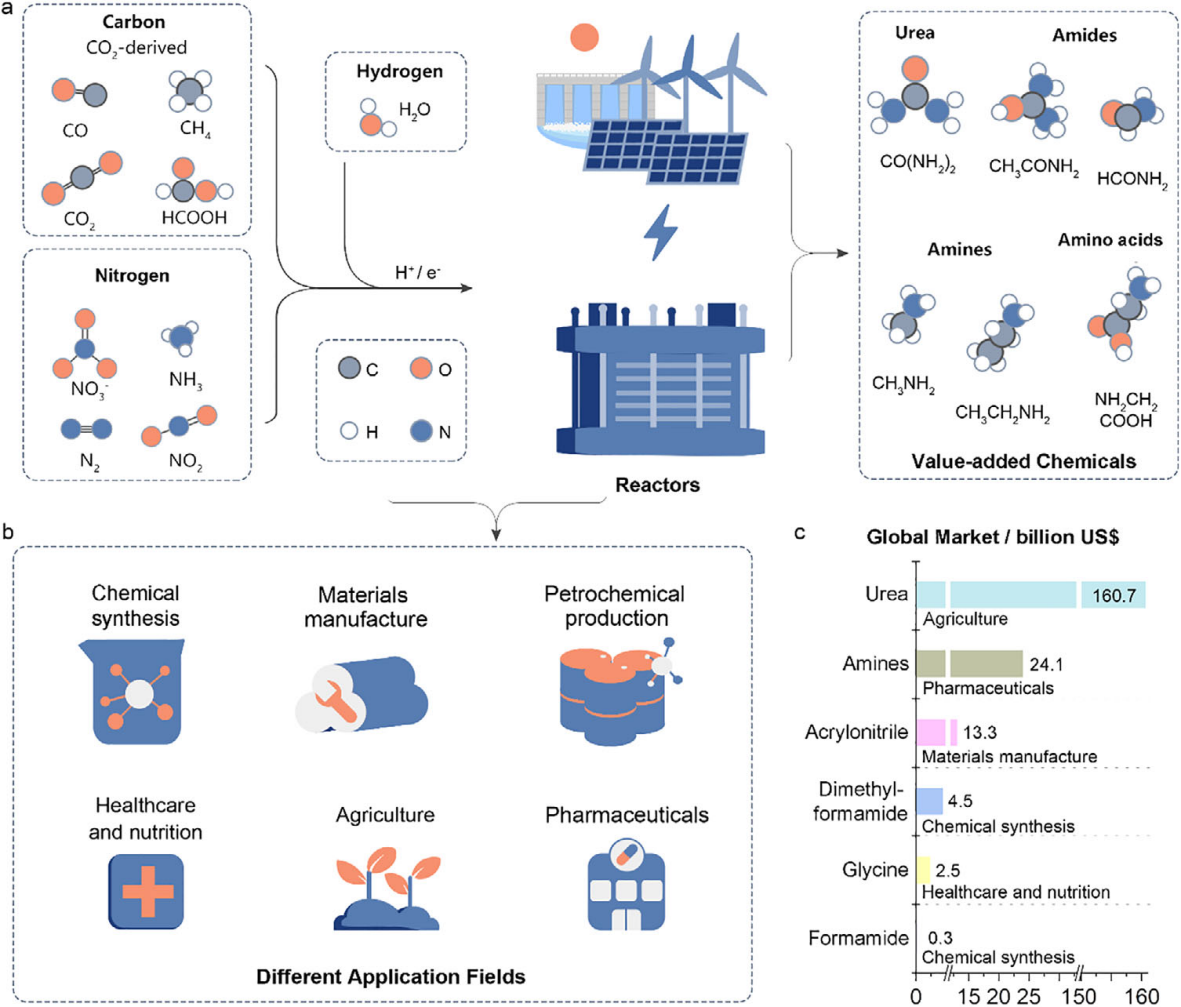
(a) Common carbon and nitrogen sources used in electrochemical C─N coupling; (b) Application areas for high‐value‐added C─N coupling products generated through electrocatalytic reactions; (c) Market size projections for various high‐value‐added C‐N coupling products.

Electrosynthesis of C─N compounds in aqueous environments can be achieved under mild conditions (room temperature and pressure) using various C sources (CO_2_, CO, etc.) and N sources (N_2_, NO_3_^−^, NO, NO_2_^−^, and NH_3_, etc.) to produce high‐value‐added products such as urea, oxime, amide, amine, and amino acids (Figure 1a, b) [26, 27, 30]. The rapid development of society has led to a consistent year‐on‐year expanding in global demand for C─N compounds (Figure 1c). The dimethylformamide market is expected to reach $4.5 billion by 2032, acrylic acid chemicals will exceed $2.5 billion by 2032, acrylonitrile chemicals will exceed $13.3 billion by 2030, and amine products are expected to exceed $24 billion by 2032 [31, 32]. Similarly, the formamide and glycine markets are expected to be close to $0.27 and $2.5 billion by 2027 and 2035, respectively [33, 34]. Urea, as an important C─N compound, is expected to have a market size of more than $160.7 billion by 2032 [35]. These small‐molecule C─N compounds serve as primary raw materials for bulk and fine chemical production, highlighting their immense commercial value and potential. As a transformative approach for synthesizing high‐value nitrogenous compounds, electrocatalysis offer a compelling technical, economic, and environmental advantages. Recent studies vividly illustrate this potential. For instance, the electrosynthesis of methylamine via nitromethane reduction on a Cu catalyst achieved an exceptional faradaic efficiency (FE) of 97% under ambient conditions, drastically lowering the energy intensity compared to conventional high‐pressure ammonolysis [36]. Similarly, the coelectrolysis of CO and NH_3_ to produce acetamide has been optimized to reach over FE (85%) at 300 mA cm^−2^ through precise pH control [37]. Advances extend to amino acid synthesis, where the co‐reduction of oxalic acid and nitrate on a structured Bi‐based electrode yielded glycine with 78.9% selectivity at 108.2 mA cm^−2^ [38]. Economically, leveraging renewable electricity can significantly reduce operating costs, making electrosynthesis routes like that for methylamine highly competitive. Environmentally, the production of one ton of acetamide via this route avoids approximately 2.5 tons of CO_2_ emissions, while the process aligns with atom economy by minimizing waste. Together, these results underscore the role of electrocatalysis in enabling efficient, selective, and sustainable synthetic pathways.

Although electrochemical synthesis offers a sustainable alternative for producing high‐value C─N compounds, its application has historically been limited to simple organic small molecules, with conversion rates and yields that lag behind traditional methods. However, recent advancements in electrocatalytic synthesis have led to the development of more efficient aqueous electrochemical coupling systems, improved economic viability, and expanded substrate versatility [36, 37]. In theory, C─N coupling can be achieved through the electrochemical coreduction of various C─ and N─containing compounds [36]. C─N coupling reactions typically involve several steps, including adsorption and activation of reactants, dissociation of N═O/N≡N/C═O bonds, proton/electron transfer, C─N bond formation and product desorption [36]. These reactions often entail the simultaneous electroreduction of CO_2_ and inorganic N sources or a two‐step mechanism combining electroreduction with chemical processes. However, the high bond energies of N═N, N═O, and C═O bonds result in slow reaction kinetics, while competing side reactions, such as hydrogen evolution reaction (HER), complicate product distribution and reduce selectivity [1, 24, 37]. The complexity of C─N coupling is further compounded by the involvement of multiple electron and proton transfers, diverse reaction intermediates, and the influence of electrocatalyst interface structure, reaction substrates, and reactor configuration [38]. Despite these challenges, the rapid progress in carbon dioxide reduction (CO_2_RR), nitrate reduction (NO_3_RR), nitric oxide reduction (NORR), and nitrogen reduction (NRR) has provided valuable insights into C─N coupling mechanisms and large‐scale synthesis, making it a hot topic in current research [39]. New mechanisms and reactor designs are continually being reported, driving the rapid advancement of C─N coupling systems. Therefore, summarizing this emerging field is crucial. In this review, we discuss recent progress in electrochemical C─N coupling reactions using various C and N sources (e.g., CO_2_, CO, N_2_, NO_x_, NH_3_, and their derivatives). We classify and analyze the electrosynthesis of high‐value products including urea, oxime, amide, amine, and amino acids, with a particular focus on their catalytic mechanisms. Additionally, we summarize and evaluate different reactor designs, including H‐cells, membrane‐based flow reactors, and membrane electrode assembly electrolyzers, highlighting their impact on C─N coupling outcomes. Finally, we highlight the significant challenges faced by C─N coupling systems, from laboratory‐scale research to large‐scale device integration, and outline future directions for this field.

**FIGURE 2:**
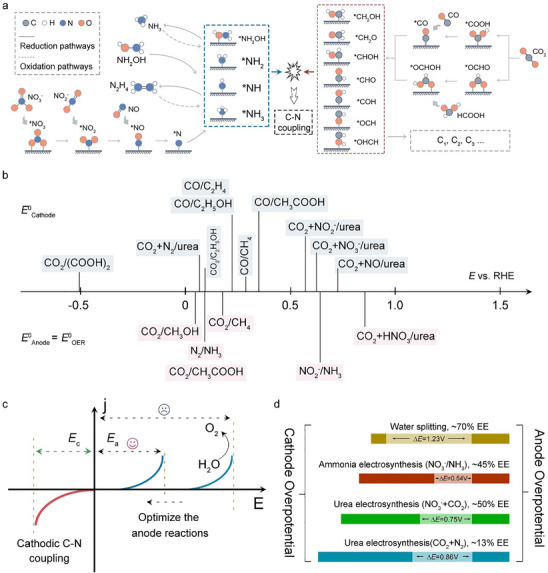
(a) Key intermediates derived from carbon and nitrogen sources in the C─N coupling reaction system; (b) Reduction potentials associated with different reduction reaction systems; (c, d) redox potential distribution for C─N coupled electrosynthesis in aqueous systems and the energy efficiency of typical reaction systems.

Under reduction potential conditions, the key reaction intermediates in the C─N coupling system primarily include hydroxylamine, adsorbed NH, NH_2_, and adsorbed NH_3_. These intermediates are generated through processes such as NO_3_RR, NORR, NRR, and NO_2_RR, as illustrated in Figure 2a. On the carbon source side, key intermediates include CH_2_OH, CH_2_O, CHOH, CHO, COH, as well as OCH and OHCH adsorbed on the O end [40, 41, 42, 43, 44]. These intermediates are primarily derived from CO_2_RR, CORR, formate reduction (COOHR), and methanol reduction (CH_2_OHRR) [45, 46, 47]. The cathode reduction potential range for these intermediates typically falls between ‐0.51 and 0.85 V versus RHE with water oxidation as the anode reaction, which is largely determined by the type of reaction precursor [1, 4, 48, 49, 50, 51, 52]. By selecting different reaction substrates, various C─N coupling products can be obtained through reaction design within specific potential ranges (Figure 2b). Notably, when water oxidation serves as the anode reaction, its high potential can limit energy efficiency as illustrated in Figure 2c [6]. To address this, alternative oxidation reaction systems can be employed to replace the oxygen evolution reaction (OER), thereby reducing overpotential and improving energy efficiency. Currently, the energy efficiency of urea synthesis (involving N_2_ and CO_2_ or CO_2_ and nitrate) in C─N coupling systems with OER as the anode is only around 50% (Figure 2d). Similarly, the energy efficiency of ammonia synthesis via NRR is approximately 45%, significantly lower than that of water splitting for hydrogen production (∼70%). This discrepancy reflects the extensive research and optimization dedicated to water splitting, while highlighting the vast untapped potential for advancing C─N coupling systems. Nitrogen sources in these reactions primarily include N_2_ and NO_x_. The reduction process involves multiple electron and proton transfers, making it highly complex. Additionally, the strong N≡N bond in N_2_ (bond energy of ∼945.1 kJ/mol) and the high energy required to break the first bond (523.3 kJ/mol), coupled with its low solubility, result in poor reactivity and slow kinetics in electrocatalytic systems using N_2_ as the nitrogen source [6]. Despite these challenges, the abundance and low cost of N_2_ make it a focal point for research into high‐value C─N coupling products. In contrast, NO_x_ species, such as nitrite (207 kJ/mol) and nitrate (204 kJ/mol), have lower dissociation energies, making C─N coupling reactions more feasible [53]. However, NO_x_ reduction involves multiple electron and proton transfers, leading to a wide range of unstable intermediates. In aqueous electrocatalytic systems, competing side reactions often result in the formation of by‐products, complicating product selectivity [54, 55]. The choice of carbon source is equally pivotal in the electrosynthesis of high‐value nitrogen‐containing compounds, such as methylamine, acetamide, and amino acids, directly determining reaction pathways and product selectivity. Employing CO_2_ requires overcoming a high‐energy activation step to form *CO intermediates, followed by coupling with nitrogen sources (e.g., NH_3_, NO_3_^−^), a pathway susceptible to hydrogen evolution yet valuable for its direct use of a sustainable feedstock [45]. In contrast, CO bypasses this initial bottleneck, enabling higher surface *CO coverage, which favors carbonylation with nitrogen species to yield amides and can promote efficient C–N bond formation via reductive amination [46]. More reactive oxygenates like ketones and aldehydes, bearing readily available carbonyl groups, allow direct nucleophilic addition or electrochemical reductive amination with amines, offering a streamlined route to amino acids in a single step. Therefore, the choice of C and N source plays a critical role in determining the reaction pathway and final product distribution. Based on the pathways involving different C and N sources, the synthesis routes and recent advancements in C─N coupling products, such as urea, amide, amine, amino acid, and oxime, are discussed in detail.

## 2 C─N Coupling Electrosynthesis of Organic Compounds

### 2.1 Urea Synthesis

**FIGURE 3:**
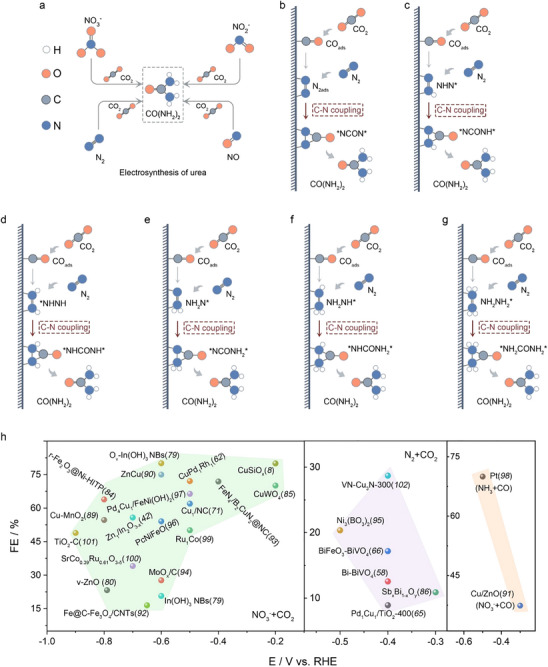
Schematic diagrams of the mechanisms involved in urea production from CO_2_ and different N‐containing sources in aqueous solutions (a). Reduction of CO_2_ and N_2_ to (b) ^*^NCON^*^, (c) ^*^NCONH^*^, (d) ^*^NHCONH^*^, (e) ^*^NCONH_2_^*^, (f) ^*^NHCONH_2_^*^, and (g) ^*^NH_2_CONH_2_^*^ intermediates. (h) Recently reported representative catalysts for urea electrosynthesis (see additional details in Table 1). The light green and pink shaded areas indicate the reaction regions for the NO_3_^−^ + CO_2_ and N_2_ + CO_2_ systems, respectively, while the light‐yellow shaded area represents the reaction regions for the NH_3_ + CO, NO_2_^−^ + CO, and NO_3_^−^ + CO systems.

In industry, urea is a downstream product of NH_3_, with approximately 80% of the world's NH_3_ further utilized for urea synthesis. This process is highly energy‐intensive, requiring conditions of 150–200 °C and 150–250 bar, resulting in an energy consumption of over 21 billion kJ ton^−1^ of urea produced [56, 57]. Given that N_2_ is the most abundant component in the atmosphere, it serves as an extremely attractive nitrogen source for urea synthesis. Since 2016, research on the electrosynthesis of urea using N_2_ and CO_2_ has advanced rapidly (Figure 3a). The key to enhancing the efficiency of urea synthesis with N_2_ lies in the adsorption and activation of gaseous reactants. To achieve this, researchers have developed various materials and methods, with new synthesis mechanisms continually being reported. Theoretical studies have revealed that the direct formation of ^*^NCON^*^ species through C─N coupling is not the sole pathway for urea production [58, 59]. Instead, the hydrogenation of N_2_ to form intermediates such as ^*^N_2_H_x_ (*x* = 1, 2, and 3) and their coupling with ^*^CO can proceed through four primary pathways (Figure 3b–g), which gradually verified by theoretical calculations.

The most widely studied pathway involves the C─N coupling to form the key intermediate ^*^NCON^*^ (Figure 3b). In this pathway, the N≡N bond in N_2_ is cleaved and adsorbed laterally on the catalyst surface, forming an N═N intermediate [60]. Simultaneously, CO_2_ molecules adsorb onto the catalytic active site, generating a ^*^CO_2_ intermediate, which is subsequently reduced to ^*^COOH and then to ^*^CO through proton‐coupled electron transfer (PCET) steps [61, 62]. The ^*^CO intermediate couples with N═N to form a tower‐shaped ^*^NCON^*^ intermediate, which undergoes further hydrogenation (4H^+^ + 4e^−^) to produce urea [63]. Notably, the *NCON* intermediate can be hydrogenated starting from one end of the N atom or through simultaneous hydrogenation at both ends (Figure 3c, d). The proposed mechanisms have been gradually validated by in situ characterization data and theoretical calculations. For instance, N_2_ forms N═N on the PdCu surface which loaded on the TiO_2_ nanosheets, while CO_2_ is reduced to ^*^CO at adjacent sites [64], exothermically coupling to form ^*^NCON^*^, which is then hydrogenated to urea. At ‐0.4 V versus RHE, the urea generation rate reaches 3.36 mmol g^−^^1^ h^−^^1^ with a FE of 8.92% [64]. Besides, the presence of space charge regions in Bi–BiVO_4_ or BiFeO_3_/BiVO_4_ mixtures verified by DFT can alter the electron density distribution at the catalyst interface, enhancing the adsorption/activation of gaseous reactants and subsequent C–N bond formation [57, 65]. Both catalysts exhibit competitive performance in 0.1 M KHCO_3_, with FE values for urea of 12.55% and 17.18%, respectively. Beyond tuning the catalyst interface structure, strategies such as regulating the spin state and d‐orbital electronic configuration of metal catalytic centers have also proven effective. Gao et al. designed Cu‐based catalysts, Cu^III^‐HHTP and Cu^II^‐HHTP, with different valence states by modulating Cu coordination in a metal‐organic framework (MOFs) [66]. This difference in electronic configuration influences electron transfer between the catalytic center and adsorbed species, leading to distinct activation pathways and catalytic activities through the DFT calculation.

Since most catalyst surfaces can reduce adsorbed ^*^N_2_ to ^*^NNH with relatively low energy input, reaction pathways involving ^*^N_2_H_x_ intermediates have been gradually verified by experimental results and theoretical revelations [10, 67, 68, 69]. The mechanisms are illustrated in Figure 3c–g. Specifically, in the N_2_‐preferential reduction pathway, adsorbed N_2_ is first converted into ^*^N_2_H_x_ intermediates via PCET steps [67]. Simultaneously, CO_2_ is reduced to ^*^CO through two PCET steps, followed by the reaction of ^*^N_2_H_x_ with ^*^CO. This process integrates NRR and CO_2_RR pathways [10, 68, 69]. As shown in Figure 3c, the urea precursor ^*^NHCON can be synthesized from ^*^NHN and ^*^CO intermediates through two pathways: (1) N_2_ + CO_2_ → ^*^NHN + ^*^CO, or (2) ^*^NHN + ^*^COOH → ^*^NHN + ^*^CO. Alternatively, after the second hydrogenation of N_2_, C─N coupling can proceed via the distal pathway (forming ^*^NH_2_N) or the alternative pathway (forming ^*^NHNH), leading to the formation of ^*^NHCONH and ^*^NH_2_CON intermediates, respectively (Figure 3d, e). Additionally, the coupling of ^*^CO with ^*^NH_2_NH or ^*^NH_2_NH_2_ can promote C─N bond formation through various pathways (Figure 3f, g). As N_2_ is difficult to activate, the electrosynthesis of urea is often compromised by competing reactions. These include the HER and the formation of organic molecules (e.g., alkanes, alcohols) from the CO_2_RR, collectively leading to low conversion rates. Although these pathways are supported by theoretical calculations, the complexity and diversity of the C─N coupling reaction system leave much controversy, necessitating further experimental and theoretical research to clarify the detailed mechanisms [70, 71].

**FIGURE 4:**
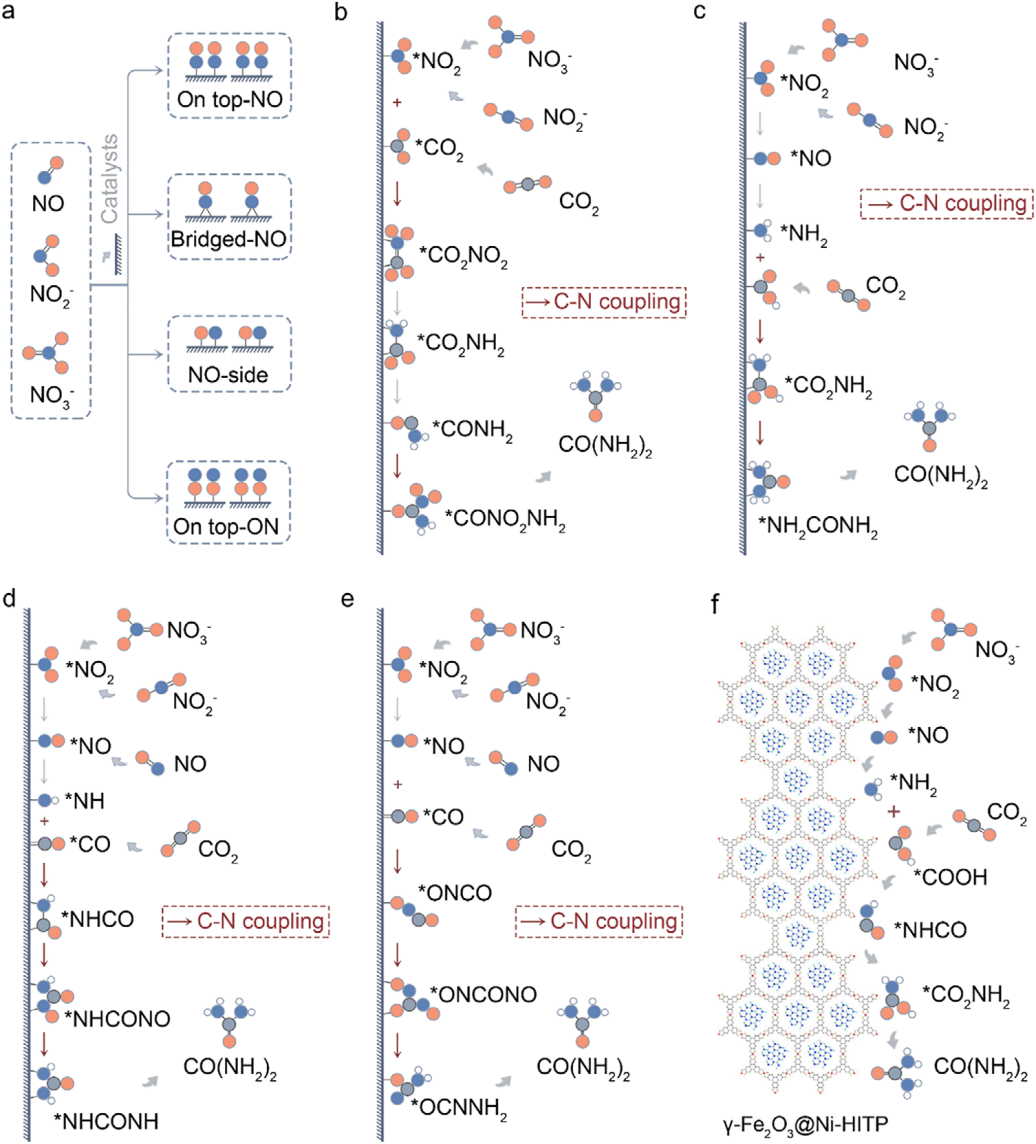
Schematic diagrams of the mechanisms involved in urea production from CO_2_ and NO_x_ in aqueous solutions, highlighting key intermediates: (a) Reduction of NO_x_ to various adsorbed ^*^NO intermediates; (b) formation of the ^*^CONO_2_NH_2_ intermediate; (c) formation of the ^*^NH_2_CONH_2_ intermediate; (d) formation of the ^*^NHCONH intermediate; (e) formation of the ^*^OCNNH_2_ intermediate; (f) the proposed reaction pathway for the co‐reduction of CO_2_ and NO_3_^−^ on the γ‐Fe_2_O_3_@Ni‐HITP catalyst suggests that when the ^*^NH_2_ intermediate is present, it preferentially couples with either the ^*^HCOO or ^*^COOH intermediates, rather than allowing these intermediates to undergo further reduction or desorption.

The practical application of N_2_ is limited by its low solubility and the high dissociation energy of the N≡N bond. In contrast, nitrite (NO_2_^−^) and nitrate (NO_3_^−^) exhibit higher solubility and lower N═O bond dissociation energy, making them promising alternative N‐sources [72]. In 1995, Furuya et al. first reported urea synthesis from CO_2_/NO_3_^−^ or CO_2_/NO_2_^−^ using Cu‐based catalysts [73]. They found that CO_2_/NO_2_^−^ yielded higher FE for urea synthesis, likely due to the lower concentration of NH_3_ precursors in the NO_3_^−^ reduction process compared to NO_2_^−^ reduction. Subsequent studies explored urea electrosynthesis using CO_2_/NO_3_^−^ or CO_2_/NO_2_^−^ systems with various metal catalysts. Interestingly, Zn‐based catalysts achieved a FE_urea_ of 50%–55% at potentials between ‐1.0 and ‐2.4 V versus RHE, demonstrating excellent catalytic potential [73]. Further developments led to electrocatalysts capable of simultaneously reducing NO_3_^−^, NO_2_^−^, or NO under ambient conditions [74]. The solution pH significantly influences the reaction, as it determines the protonation state of NO_3_^−^ and NO_2_^−^. NO_x_ species adsorb onto the catalyst surface and dissociate to form the key intermediate ^*^NO, which dictates reaction selectivity [74]. ^*^NO can either be hydrogenated to form ^*^NOH, ^*^HNO, or ^*^HNOH before N─O bond dissociation or directly dissociate into ^*^O and ^*^N, ultimately forming NH_3_, NH_4_^+^, or the byproduct N_2_H_4_ (Figure 4a). Notably, ^*^NO can react with NO_2_^−^ to form ^*^N_2_O, leading to unwanted N_2_ production [75]. The presence of N, O‐containing intermediates can be verified experimentally using in situ spectroscopy. To provide a clearer comparison of urea electrosynthesis across various catalytic systems, the key parameters of different systems were summarized and analyzed, as presented in Figure 3h and Table 1. The results reveal that urea electrosynthesis based on the co‐reduction of NO_3_^−^ and CO_2_ exhibits significantly higher reaction selectivity (FE) and yield compared to other reaction system (N_2_ + CO_2_, NH_3_ + CO, NO_2_^−^ + CO, and NO_3_^−^ + CO), demonstrating that the superior performance highlights its strong potential for practical industrialization. Specifically, designing C─N coupling catalysts requires suppressing competing reactions like HER to improve selectivity and efficiency [19, 21]. The {100}‐faceted In(OH)_3_ electrocatalyst suppresses HER by capturing electrons with adsorbed CO_2_, transforming the *n*‐type surface to *p*‐type, achieving 53.4% FE_urea_ and 82.9% nitrogen‐to‐urea conversion at ‐0.6 V via NO_3_^−^ and CO_2_ coupling [76]. Atomic defect engineering on oxygen‐deficient In(OH)_3_ nanobelts were also constructed to optimize intermediate adsorption (^*^CO_2_NO_2_, ^*^CO_2_NH_2_) and enhance C−N coupling, reaching a record 80.1% FE_urea_, 2.9 times higher than pristine In(OH)_3_ [77]. To date, two mechanisms have been proposed by the summary of experimental results for urea synthesis: (1) the Eley‐Rideal mechanism, where surface‐bound ^*^N_1_ species react with gaseous CO_2_ to form a C−N bond (^*^N_1_ + CO_2_); and (2) the Langmuir‐Hinshelwood mechanism, where adsorbed ^*^C_1_ and ^*^N_1_ species integrate to form a C−N bond (^*^N_1_ + ^*^COOH/^*^CO) [78, 79]. The adsorption configuration of NO_x_ on the catalyst surface, including on‐top‐ON, NO‐side, bridged‐NO, and on‐top‐NO (Figure 4a), can influence the FE or yield [78]. In the Eley‐Rideal mechanism, ^*^NO_2_ and ^*^CO_2_ intermediates form ^*^CO_2_NO_2_ (Figure 4b), which undergoes six PCET steps to generate ^*^CO_2_NH_2_ [78, 79]. Protonation of ^*^CO_2_NH_2_ to ^*^COOHNH_2_ is considered the rate‐determining step. Subsequent C─N coupling forms ^*^CONH_2_, followed by a second coupling step to produce urea via the ^*^CONO_2_NH_2_ intermediate (Figure 4c). In the Langmuir‐Hinshelwood mechanism, ^*^NH_2_ and ^*^COOH intermediates couple to form ^*^CO_2_NH_2_, followed by a second C─N coupling to produce ^*^NH_2_CONH_3_, ultimately yielding urea [78, 79]. This pathway has been reported on oxygen‐deficient ZnO surfaces [78]. Additionally, ^*^NH and ^*^CO can bond on Fe‐Ni pairs to form ^*^NHCO, promoting urea synthesis (Figure 4d) [70]. Other studies have demonstrated urea synthesis on Te‐doped Pd nanocrystals (Te‐Pd NCs) via ^*^CO and ^*^NH_2_ coupling [80]. Interestingly, unlike oxygen‐deficient ZnO, CeO_2_ with oxygen vacancies facilitates C─N coupling between ^*^NO and ^*^CO as shown in Figure 4e [27]. Besides, a novel strategy encapsulated ultrasmall γ‐Fe_2_O_3_ nanoparticles (<2 nm) within the pores of a conductive metal–organic framework (γ‐Fe_2_O_3_@Ni‐HITP), Ni‐HITP (HITP = 2,3,6,7,10,11‐hexaaminotriphenylene), demonstrated exceptional electrocatalytic performance for urea synthesis [81]. It achieved a FE_urea_ of 67.2% and a remarkable urea yield rate of 7.7 mg h^−1^ cm^−2^. Mechanistic studies revealed that Fe(III) ions in the γ‐Fe_2_O_3_ nanoparticles play a critical role, facilitating the generation of key intermediates (^*^NH_2_ and ^*^COOH). Additionally, adjacent Fe(III) ion pairs in the γ‐Fe_2_O_3_ nanoparticles serve as highly active sites, promoting C–N coupling between ^*^NH_2_ and ^*^COOH to form the crucial intermediate ^*^CONH_2_ (Figure 4f). This synergistic mechanism underpins the outstanding performance of γ‐Fe_2_O_3_@Ni‐HITP for urea production. Significant progress has been made in the electrosynthesis of urea, particularly in the development of novel catalysts. However, advancing this field further requires the design of highly stable and active catalysts, as well as a deeper understanding of the C–N coupling mechanism to enhance selectivity and efficiency. The higher reducibility of NOx compounds compared to N_2_ introduces a significant competing pathway in urea electrosynthesis. When NO species serve as the nitrogen source, the system must contend not only with HER and the reduction of the carbon source to alkanes/alcohols, but also with the substantial generation of NH_3_ as a primary side reaction. Although urea synthesis is established and mechanistic understanding continues to advance, current research in electrocatalytic urea synthesis predominantly focuses on metrics such as FE and yield under low current densities. For industrial viability, emphasis must shift toward achieving high single‐pass conversion, substantial product concentration, and long‐term operational stability under industrially relevant current densities (>200 mA cm^−2^). These performance parameters will constitute the critical research frontier for the next phase of electrosynthetic urea development.

**TABLE 1:** Heterogeneous catalysts used for electrosynthesis of urea.

Catalyst	Reactants	Media	Key C─N intermediates	FE	Potential (V_RHE_)^a^	Yield
v‐ZnO [78]	NO_3_^−^+CO_2_	0.2 M NaHCO_3_	^*^NH_2_+^*^COOH	23.26%	−0.79 V	16.56 µmol h^−1^
r‐Fe_2_O_3_@Ni‐HITP [81]	NO_3_^−^+CO_2_	0.1 M KHCO_3_	^*^NH_2_+^*^COOH	63.9%	−0.8 V	7.7 mg h^−1^ cm^−2^
Cu_1_/NC [69]	NO_3_^−^+CO_2_	0.1 M KHCO_3_	^*^NOOH+^*^CO	62%	−0.5 V	596.1 µg mg^−1^ h^−1^
CuWO_4_ [82]	NO_3_^−^+CO_2_	0.1 M KNO_3_	^*^NO_2_+^*^CO	70.1%	−0.2 V	98.5 µg mg^−1^ h^−1^
Ru_1_Cu SAA [43]	NO_2_^−^+ CO	1 M KOH	^*^NOOH+^*^CO	45.65%	−0.5 V	2483.77 µg h^−1^ mg^−1^
O_v_‐In(OH)_3_ NBs [77]	NO_3_^−^+CO_2_	1 M KNO_3_	^*^NO_2_+^*^CO_2_	80.1%	−0.6 V	–
In(OH)_3_ NBs [77]	NO_3_^−^+CO_2_	1 M KNO_3_	^*^NO_2_+^*^CO_2_	20.7%	−0.6 V	–
Bi‐BiVO_4_ [57]	N_2_ + CO_2_	0.1 M KHCO_3_	^*^N = N + ^*^CO	12.55%	−0.4 V	5.9 mmol g^−1^ h^−1^
BiFeO_3_‐BiVO_4_ [65]	N_2_ + CO_2_	0.1 M KHCO_3_	^*^N = N + ^*^CO	17.18%	−0.4 V	4.94 mmol h^−1^ g^−1^
Sb_x_Bi_1‐x_O_y_ [83]	N_2_ + CO_2_	0.5 M K_2_SO_4_	^*^N = N + ^*^CO	10.9%	−0.3 V	307.97 µg h^−1^ mg_cat._^−1^
Cu_1_CeO_2_ [84]	NO_3_^−^+CO_2_	0.1 M KHCO_3_	^*^NO +^*^CO	–	−1.6 V	52.84 mmol h^−1^ g_cat._^−1^
CuBi_2_O_4_ [85]	N_2_ + CO_2_	H_2_O	^*^N = N + ^*^CO	–	–	2.7 mmol g^−1^ h^−1^
Cu^III^‐ HHTP [66]	N_2_ + CO_2_	0.1 M KHCO_3_	^*^NH_2_+^*^CO	23.09%	−0.6 V	7.78 mmol h^−1^ g^−1^
Cu─MnO_2_ [86]	NO_3_^−^+CO_2_	0.05 M KNO_3_ + 0.1 M KHCO_3_	^*^NH+^*^CO	54.7%	−0.8 V	116.7 mmol h^−1^ g_cat_^−1^
CuSiO_x_ [8]	NO_3_^−^+CO_2_	0.1 M KNO_3_ +0.1 M KHCO_3_	*NO_2_ + *CO	80%	−0.2 V	1606.1 µgh^−1^ mg_cat_^.‐1^
CuPd_1_Rh_1_ [61]	NO_3_^−^+CO_2_	0.1 M KNO_3_ +0.1 M KHCO_3_	^*^NO_2_+^*^CO_2_	72.1%	−0.5 V	53.2 mmol h^−1^ g_cat_^−1^
ZnCu [29]	NO_3_^−^+CO_2_	0.1 M KHCO_3_ + 500 ppm KNO_3_	^*^NO_2_+^*^CO_2_	75%	−0.6 V	1.7 µmol h^−1^ cm^−2^
Cu/ZnO [88]	NO_3_^−^+CO_2_	0.1 M KNO3	^*^NH_2_+^*^CO	37.4%	−0.3 V	3.2 µmol h^−1^ cm^−2^
Fe(a)@C‐Fe_3_O_4_/CNTs [89]	NO_3_^−^+CO_2_	0.1 M KNO_3_	^*^NH_2_+^*^CO	16.5%	−0.65 V	1341.3 µgh^−1^ mg_cat_^−1^
FeN_4_/B_2_CuN_2_@NC [90]	NO_3_^−^+CO_2_	0.1 M KHCO_3_ +0.1 M KNO_3_	^*^NO_2_+^*^CO_2_	71.9%	−0.4 V	2.07 mg h^−1^ mg_cat._^−1^
FeNiDASC [70]	NO_3_^−^+CO_2_	0.1 M KHCO_3_ +50 mM KNO_3_	^*^NH+^*^COOH	17.8%	−1.5 V	20.2 mmol h^−1^ g^−1^
MoO_x_/C [91]	NO_3_^−^+CO_2_	0.1 M KNO_3_	^*^NO_2_+^*^CO_2_	27.7%	−0.6 V	1431.5 µgh‐^1^ mg_cat._^−1^
Ni_3_(BO_3_)_2_ [92]	N_2_+ CO_2_	0.1 M KHCO_3_	^*^N = N + ^*^CO	20.36%	−0.5 V	9.70 mmol h^−1^ g_cat._^−1^
PcNi‐Fe‐O [93]	NO_3_^−^+CO_2_	0.1 M KNO_3_	^*^NH+^*^CO_2_	54.1%	−0.6 V	2.03 gh^−1^ g_cat._^−1^
Pd_1_Cu_1_/TiO_2_‐400 [64]	N_2_+ CO_2_	H_2_O	^*^N = N + ^*^CO	8.92%	−0.4 V	3.36 mmol g–^1^ h–^1^
Pd_4_Cu_1_/FeNi(OH)_2_ [43]	NO_3_^−^+CO_2_	0.1 M KHCO_3_+0.1 M KNO_3_	^*^NH_2_+^*^CO	66.4%	−0.5 V	436.9 mmol g_cat.–_^1^ h–^1^
Pt [94]	NH_3_+ CO	0.1 M KOH + (NH_4_)_2_SO_4_	^*^N+^*^CO	70%	−0.5 V	100 mmol h^−1^ g_cat_^.−1^
Ru_1_Co [95]	NO_3_^−^+CO_2_	0.1 M KNO_3_ +0.1 M KHCO_3_	^*^NH_2_+^*^CO	50.1%	−0.5 V	22.34 mmol h^−1^ g^−1^
SrCo_0.39_Ru_0.61_O_3‐δ_ [96]	NO_3_^−^+CO_2_	0.1 M KNO_3_	^*^NH_2_+^*^CO	34.1%	−0.7 V	1522 µgh^−1^mg_cat._^−1^
TiO_2_‐C [97]	NO_3_^−^+CO_2_	0.1 M KNO_3_	^*^NH_2_+^*^COOH	48.88%	−0.9 V	43.37 mmol g^−1^h^−1^
VN‐Cu_3_N‐300 [98]	N_2_+CO_2_	0.1 M KHCO_3_	^*^N_2_+^*^CO	28.7%	−0.4 V	81 µg h^−1^ cm^−2^
Zn_1_/In_2_O_3‐x_ [42]	NO_3_^−^+CO_2_	0.1 M KNO_3_ + 0.1 M KHCO_3_	^*^NH_2_+^*^CO	55.8%	−0.7 V	41.6 mmol h^−1^ g^−1^
Zn nanobelts [99]	NO+CO_2_	0.2 M KHCO_3_	^*^NH_2_+^*^COOH	11.26%	−0.92 V	15.13 mmol h^−1^g^−1^

^a^ Unless noted otherwise, the working potentials are given relative to the RHE, with the RHE serving as the reference for measuring the FE.

### 2.2 Amines Synthesis

Common amine compounds, such as monomethylamine, dimethylamine, and trimethylamine, are essential industrial chemical intermediates widely used in the production of pesticides, pharmaceuticals, solvents, and water treatment agents. Industrially, most amines are synthesized through the reaction of NH_3_ with corresponding alcohols on dehydration catalysts [25, 100]. The electrochemical C─N coupling of nitrogen‐containing compounds and carbon sources offers a safer, greener, and more sustainable alternative for amine synthesis [101].

**FIGURE 5:**
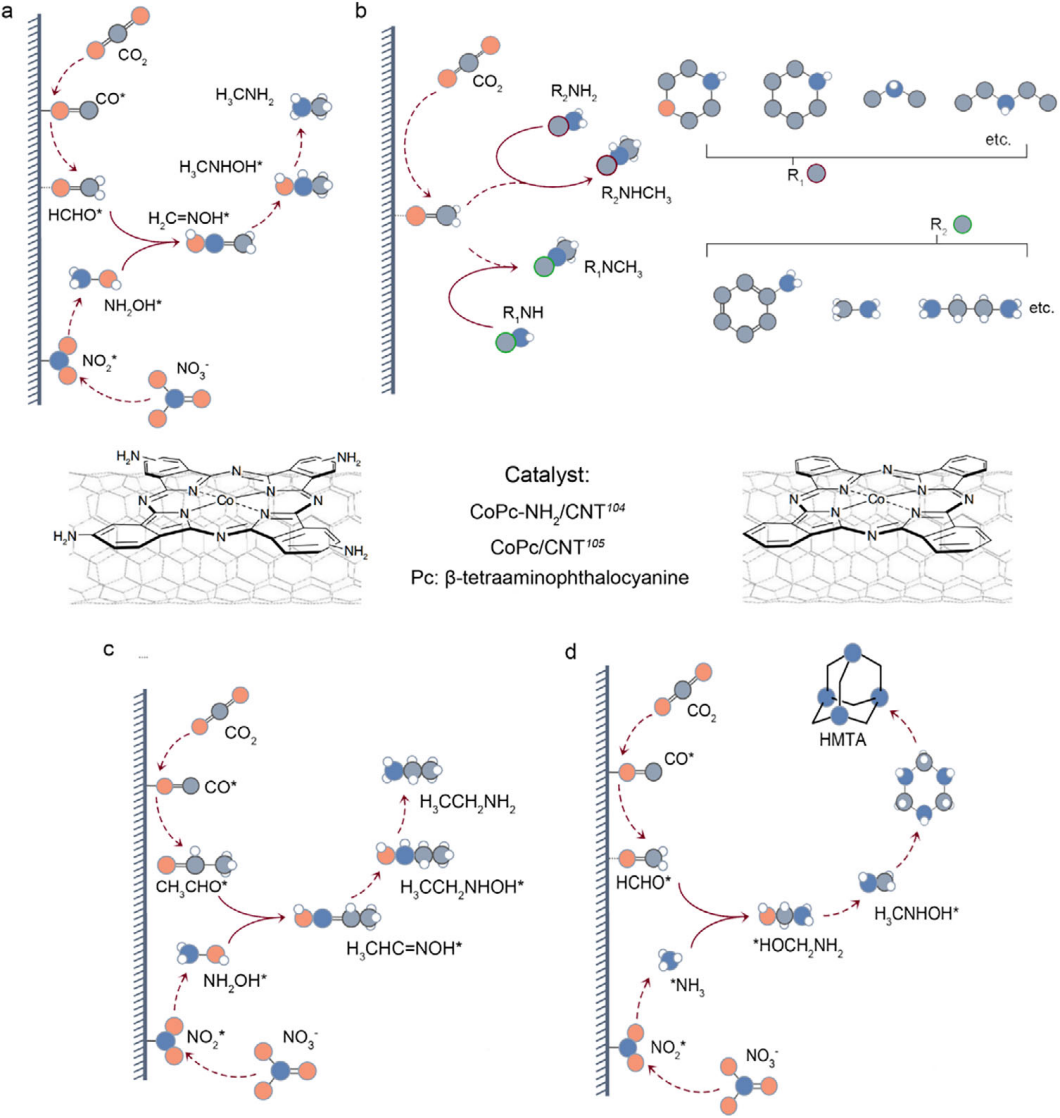
Schematic diagrams of amine synthesis in aqueous solutions, highlighting key intermediates: (a) ^*^H_2_C═NOH (formaldehyde oxime); (b) C─N coupling of aldehyde intermediates (generated via CO_2_ reduction) with alkylamines to produce amines. (c) ^*^H_3_CHC═NOH (acetaldehyde oxime); (d) ^*^HOCH_2_NH_2_ (hydroxy methylamine).

The electrochemical C─N coupling synthesis of amine compounds involves multiple electron and proton transfer processes, with the formation of aldehyde intermediates often serving as a key step in the production of amine products [25, 100, 101]. Wu et al. pioneered the electrocatalytic synthesis of methylamine (CH_3_NH_2_) using carbon nanotube‐supported cobalt β‐tetra amino phthalocyanine (CoPc‐NH_2_/CNT) as a catalyst, coreducing CO_2_ and NO_3_^−^. The process involves an eight‐step catalytic cascade, with each CH_3_NH_2_ requiring the transfer of 14 electrons and 15 protons. [25] A key step is the rapid nucleophilic attack of the intermediate ^*^NH_2_OH on ^*^HCHO to form ^*^H_2_C─NOH (formaldehyde oxime) which can be identified by in situ infrared spectroscopy. The ^*^H_2_C═NOH is then electrochemically reduced to yield CH_3_NH_2_ (Figure 5a). Subsequently, the reaction scope was further expanded to include various N‐containing reagents, such as primary amines, secondary amines, alkylamines, and aromatic amines (Figure 5b). At ‐0.93 V versus RHE, it achieved FEs of 8.6% for CH_2_NNH_2_, 0.6% for CH_3_NHNH_2_, 2.0% for CH_3_NH_2_, and 6% for N‐methylaniline [100]. The key intermediate *HCHO undergoes chemical condensation with N nucleophiles, enabling the synthesis of different alkylamines by modifying the C chain ligands [100]. In another study, oxide‐derived Cu nanoparticles were used to reduce NO3^−^ and CO_2_ to ethylamine [101]. After 5 h of electrolysis at ‐1.0 V versus RHE, the FE for ethylamine was 0.3%, limited by competing processes such as the self‐reduction of acetaldehyde and hydroxylamine [101]. The reaction mechanism resembles that of CH3NH2 synthesis, with acetaldehyde oxime (CH3CH═NOH) as the key intermediate (Figure 5c). CO_2_ is electrochemically reduced to acetaldehyde, which reacts with *NH2OH (generated from NO3^−^ reduction) to form acetaldehyde oxime, ultimately yielding ethylamine [101]. Beyond simple alkylamines, hexamethylenetetramine (HMTA) also can be synthesized through the electrocatalytic coupling of NO3^−^ and HCHO using electrochemically oxidized‐derived Cu (e‐OD‐Cu) as the catalyst [102]. At ‐0.3 V versus RHE, the yield and FE for HMTA reached 76.8% and 74.9%, respectively. The reaction involves both electrochemical and chemical processes: NO3^−^ is reduced to *NH3, which then reacts with *HCHO to form key intermediates through two pathways, ultimately combining to produce HMTA (Figure 5d). This work demonstrates an electrochemical strategy for constructing multiple C−N bonds efficiently [102]. Unlike the strategies mentioned above, researchers have developed a “one‐pot, two‐step” electrochemical reductive amination method. This approach employs NO2^−^ as the N source and aldehydes or ketones as the carbon source, with both steps carried out in a neutral phosphate buffer solution. First, a Pd catalyst couples NO2^−^ with the aldehyde or ketone to form an oxime intermediate, which is then reduced to the corresponding amine using a Pb/PbO catalyst. This strategy eliminates the need for traditional amine precursors, chemical reductants, and organic solvents, resulting in a greener, safer, and more efficient process. Using acetone as a model substrate, isopropyl amine was synthesized with 50% Faradaic efficiency, 85% carbon selectivity, and 34% nitrogen selectivity [103]. It should be noted that the current densities employed for amine synthesis in the aforementioned studies are relatively low. Furthermore, the long‐term stability of the catalysts remains unverified, presenting significant barriers to practical application. To enable scalable production, atomically dispersed Ru‐doped Cu alloy electrodes (Ru_1_Cu) were fabricated. In the model reduction of *p*‐nitrophenol to *p*‐aminophenol, the mechanism shifted from conventional PCET to hydrogen atom transfer (HAT). Across a broad potential window (∼500 mV), over the full pH range (0–14), and at varying substrate concentrations (12.5–250 mM), the system consistently delivered >99% yield, selectivity, and FE, even at industrial‐grade current densities (∼350 mA cm^−^^2^). Continuous operation in a flow cell for 1060 h showed no significant structural degradation of the catalyst and stable current density. A single‐cell operation successfully produced *p*‐aminophenol on the kilogram scale (0.511 kg), underscoring its strong potential for industrial implementation [104]. Additionally, a synergistic chemical microenvironment and electrochemical microenvironment regulation strategy has been reported. Using a Cu catalyst, by modulating the local nitrite/benzaldehyde concentration at the electrode interface and employing pulse electrolysis in a flow reactor, the Faradaic efficiency for benzylamine synthesis was significantly enhanced, reaching up to 65.2% [105].

### 2.3 Amino Acids Synthesis

**FIGURE 6:**
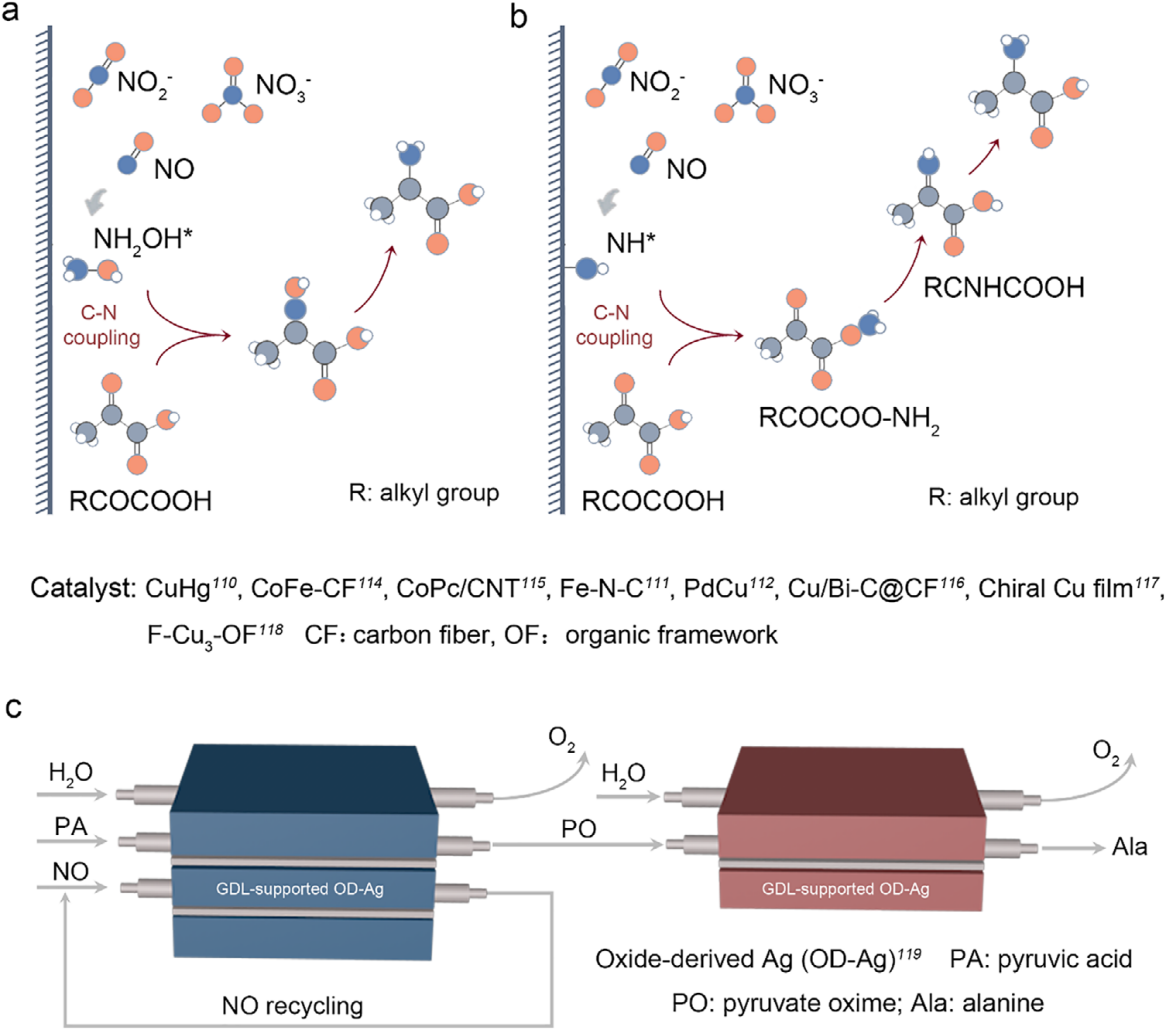
Synthesis of amino acids using NO_x_ and α‐keto acids via key intermediates of (a) hydroxylamine and (b) NH_x_ pathway. (c) The two‐pot route to improve the yield of alanine.

Amino acids are widely used in daily life and the pharmaceutical industry. Traditional synthesis methods often involve complex procedures or toxic raw materials, leading to inefficiency, cumbersome purification processes, and high energy consumption [106]. Recent studies have demonstrated the feasibility of synthesizing amino acids using NO_x_ as N source and various C sources [107]. Based on the key intermediates involved, these reactions can be classified into two categories: (1) C─N coupling reactions with hydroxylamine as the key intermediate (Figure 6a), and (2) C─N coupling reactions with NH_x_ as the key intermediate and a carbon source (Figure 6b).

Recently glycine was synthesized via the co‐reduction of NO_3_^−^ and oxalic acid (OA) using a CuHg catalyst [105]. In this process, oxalic acid is reduced to electrophilic glyoxylic acid, while NO_3_^−^ is reduced to nucleophilic hydroxylamine by the analysis of in situ infrared spectroscopy. These intermediates undergo C─N coupling to form acetaldehyde oxime, which is further reduced to glycine with a FE of up to 43.1% [108]. This study overcomes the limitation of non‐electrochemical reductive amination, which typically requires active aldehydes/ketones and amines [108]. The generation of hydroxylamine and glyoxylic acid is critical for C─N bond formation. Similarly, atomically dispersed Fe─N─C catalysts were used to co‐reduce OA with NO_3_^−^ or NO_x_, achieving glycine synthesis [109]. Based on the DFT calculation, the synergistic effect of the Fe─N─C structure and pyrrole N sites in the catalyst facilitates the formation of glyoxylic acid (GX), which couples with NH_2_OH to form GX oxime [109]. This intermediate is then hydrogenated to glycine, with the Fe─N─C structure lowering the energy barrier for ^*^HOOCCH_2_NH_2_ intermediate formation, enhancing glycine electrosynthesis [109]. In another study, PdCu nanobead wires were employed as electrocatalysts for the cascade synthesis of alanine from biomass‐derived pyruvate (PA) and NO_3_^−^ in a flow cell device [110]. The process involves a series of electrochemical steps verified by DFT calculation: Cu promotes the reduction of NO_3_^−^ to NH_2_OH, which chemically couples with PA to form acetone oxime. Pd then facilitates the electrochemical reduction of acetone oxime to alanine, achieving a yield of 54.8% at ‐0.3 V versus RHE [110]. The coupling of ^*^NH_2_OH with PA inhibits its reduction to NH_3_, enhancing alanine production [110]. Atomically dispersed Fe─N─C was also used to electro synthesize various α‐amino acids from nitric oxide (NO) and α‐keto acids [111]. At ‐0.6 V versus RHE, valine was produced with a yield of 32.1 µmol mg_cat_^−1^ and a selectivity of 11.3% [110]. NO, NO_2_, and NO_3_^−^ were all found to serve as N sources, reducing to hydroxylamine, which nucleophilically attacks α‐keto acids to form oximes and then hydrogenated to yield amino acids [110]. It should be noted that when employing these N sources, parasitic reactions, notably the HER and NH_3_ formation, adversely affect both the FE and the overall conversion rate of the target synthesis. Similarly, a self‐standing carbon fiber membrane with CoFe alloy (CoFe‐SSM) converted NO and α‐keto acids into α‐amino acids, with leucine production reaching 115.4 µmol h^−^^1^ and an FE of 32.4% [112]. This method was extended to other nitrogen sources (NO_2_^−^, NO_3_^−^, or NO_2_), successfully synthesizing 13 amino acids [112]. Besides, carbon nanotube‐supported cobalt phthalocyanine (CoPc/CNT) catalysts have also demonstrated efficient amino acid synthesis from NO_3_^−^ and α‐keto acids (e.g., alanine, glutamate, glycine, leucine, and valine) [112]. The CoPc catalyst reduces NO_3_^−^ to NH_2_OH, which attacks the α‐carbon of α‐keto acids to form oximes [113]. The CNT then reduces these oximes to amino acids, showcasing the dual‐functional synergy of CoPc/CNT in achieving efficient C─N coupling [113]. Additionally, a copper‐bismuth bimetallic catalyst (Cu/Bi‐C@CF) derived from a metal‐organic framework (MOF) array was used to synthesize glycine from glyoxylate and NO_3_^−^ [114]. The catalyst achieved 89% selectivity and 65.9% FE for glycine. The reaction pathway involves NO_3_^−^ reduction to ^*^NH_2_OH, followed by oxime formation and sequential deoxygenation/hydrogenation to glycine [114]. The introduction of Bi in the catalyst reduces Cu binding energy, enhancing NH_2_OH selectivity and promoting oxime formation [114]. These studies highlight that the generation of hydroxylamine and its coupling with C sources to form oximes is a key step in amino acid synthesis.

Unlike C─N coupling systems that rely on hydroxylamine intermediates, amino acids can also be synthesized through reactions between C sources and NH_x_ intermediates [115, 116]. This mechanism expands the possibilities for synthesizing multi‐carbon amino acids. For instance, C_3_^+^ amino acids were successfully synthesized via electrocatalysis using chiral Cu films (CCFs) as catalysts with CO_2_ and NH_3_ as raw materials [115]. Experimental and theoretical studies revealed that the adsorption of histidine induces the formation of chiral kink sites on the CCF surface [115]. These sites, through their geometric arrangement, limit the configuration of C_3_^+^ intermediates on the catalyst surface, reducing the reaction energy barrier for C─N coupling through the DFT calculation [115]. The formation of H_2_CO‐CO intermediates was identified as the rate‐determining step for C_3_^+^ product generation [115]. Additionally, a trinuclear Cu cluster‐containing perfluoroalkyl organic framework (F‐Cu_3_‐OF) was designed for the efficient electrosynthesis of various amino acids, including glycine, alanine, leucine, valine, and phenylalanine, from NO_3_^−^ and ketoacids [116]. The hydrophobic perfluoroalkyl groups within the F‐Cu_3_‐OF pores prevent competitive HER by repelling polar H^+^ while enhancing the adsorption of less polar NO_3_^−^ and ketoacids on active sites [116]. The H bonding interaction between ^*^CH_3_CNHCOO^−^ and ^*^NH_2_ intermediates facilitates a thermodynamically spontaneous C─N coupling reaction, forming ^*^CH_3_CNHCOO^−^, which is then reduced to alanine [116]. Despite significant progress in the electrosynthesis of amino acids, most research has focused on catalyst design, while large‐scale production remains a major challenge. To address this, innovative solutions such as cascade reaction devices are being developed. One‐pot synthesis has been reported for synthesizing alanine on low‐coordinated silver (OD‐Ag) using NO and biomass‐derived PA. [117]. The system achieved an FE of 17.0% and a yield of 11.45 mmol h^−^^1^ g_cat_^−1^. Experimental and theoretical studies showed that NO is reduced to ^*^NH_2_OH, which spontaneously condenses with PA to form pyruvic acid oxime [117]. This intermediate is then reduced to alanine (Figure 6c). However, the system faces challenges due to the rapid generation of pyruvic acid oxime (PO) and its slow reduction, leading to byproduct formation and reduced yields. To overcome this, a spatially decoupled two‐compartment flow reactor system was reported, achieving a total FE of 75%, >98% purity, >90% yield, and gram‐scale alanine production at 100 mA cm^−2^ [117]. This method can also be extended to the production of glycine, aspartic acid, and glutamic acid, offering valuable insights for the scalable synthesis of amino acids. While cascade reactors have been engineered for amino acid synthesis, yields and process economics at low current densities remain insufficient for viable production. Advancing the industrialization of electro synthesized amino acids will therefore, require concurrent innovation in both catalyst and reactor design.

### 2.4 Amides Synthesis

Amides play a pivotal role in chemical science due to their ubiquitous presence in biological systems and the pharmaceutical industry. They form the backbone of peptides, proteins, and numerous biomolecules, making them indispensable in medicine. It is estimated that amide bond formation is the most common reaction in pharmaceutical synthesis, with approximately 25% of marketed drugs (and two‐thirds of candidate drugs) containing at least one amide bond [118].The resurgence of electrosynthesis has spurred the development of more sustainable methods for amide synthesis, including electroreduction and electrooxidation approaches, which hold great promise for the future of synthetic chemistry [119, 120].

**FIGURE 7:**
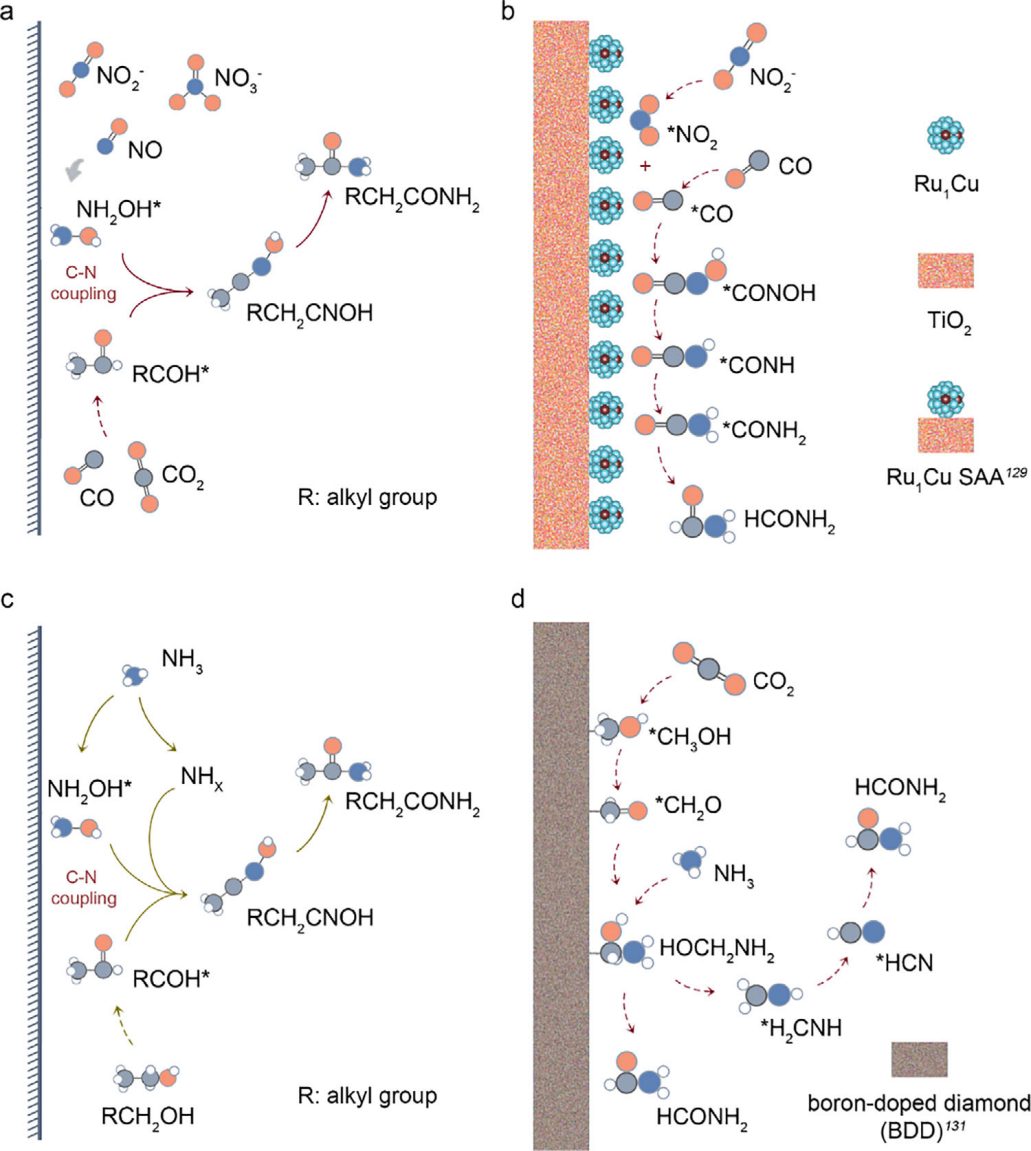
(a) Synthesis of amino acids via electroreduction using NO_x_ and CO_x_ as nitrogen and carbon sources, respectively; (b) HCONH_2_ electrosynthesis from CO and NO_2_^−^ based on TiO_2_‐Ru_1_Cu catalyst. (c) Synthesis of amino acids via electrooxidation using NH_3_ and alcohols as N and C sources. (d) Large‐Scale synthesis of HCONH_2_ from CO_2_ and NH_3_ with the BDD as catalyst.

The coupling of C (^*^CHO or ^*^CO) intermediates with N intermediates (^*^NH_x_ or ^*^NH_2_OH) is critical for primarily electrochemical synthesis of amides (Figure 7a). Cu nanoparticles were early reported to be used to co‐electro reduce NH_3_ and CO to produce acetamide for electrochemical amide synthesis [121]. The reaction proceeds via nucleophilic addition of NH_3_ to a surface‐bound ionone intermediate as the experimental results. This mechanism has been successfully extended to synthesize a range of amides, including N‐methylacetamide, N‐ethylacetamide, *N,N*‐dimethylacetamide, and acetic monoethanolamide [121]. A low coordination Cu catalyst was further prepared for converting formate and nitrite to form amide by the electrochemical process [122]. The key step involves the coupling of ^*^CHO and ^*^NH_2_ intermediates to form the C─N bond, enabling high‐performance formamide synthesis [122]. This strategy was also applied to synthesize acetamide using acetic acid as the C source and NO_3_^−^ as N source [122]. As the composition varies, diverse carbon‐containing intermediates can also emerge on the Cu catalyst surface. Ru single atom doped into Cu nanoclusters supported on TiO_2_ was developed to achieve formamide synthesis from CO and NO_2_^−^ [123]. Based on the DFT calculation, CO was dissociated and adsorbed at Cu sites, while NO_2_^−^ was deoxygenated and hydrogenated at Ru sites to form the key intermediate ^*^NH_2_. The coupling of ^*^CO and ^*^NH_2_ enabled efficient formamide electrosynthesis [123]. Aside from the beneficial catalytic impact of metallic Cu, CuO_x_ also exhibit outstanding catalytic properties. By incorporating *p*‐block metal oxides into CuO_x_, CuO_x_/BiO_x_ catalyst was created to enhance the electrocatalytic C─N coupling capability [124]. This catalyst co‐electro reduced CO_2_ and NO_x_^−^ to formamide (HCONH_2_) in a flow electrolyzer, achieving a yield of 134 ± 11 mmol h^−^^1^ g_cat_^−1^ (FE of 4.8 ± 0.4%) at a cell potential of ‐3.0 V, the highest reported yield for HCONH_2_ synthesis in a flow system [124]. The catalyst provides abundant active sites for reactant adsorption and activation, promoting the formation of ^*^CHO and ^*^NH_2_ intermediates, which couple to form formamide [124]. In contrast to the above mechanisms, Ma et al. utilized a gas diffusion electrode (GDE) loaded with small Cu nanoparticles to electrochemically convert CO_2_ and NO_2_^−^ to acetamide under alkaline conditions [125]. Key intermediates, acetaldehyde and hydroxylamine (NH_2_OH), undergo nucleophilic addition to form acetaldehyde oxime, which is dehydrated to acetonitrile in an alkaline electrolyte and subsequently hydrolyzed to acetamide. This mechanism was also applied to synthesize phenylacetamide from phenylacetaldehyde and NH_2_OH [125]. Interestingly, CO_2_ does not always need to be reduced to ^*^CHO or ^*^CO to participate in C─N coupling. Pd nanoparticle‐loaded Cu nanosheets enabled the electrosynthesis of *N, N*‐dimethylformamide (DMF) from CO_2_ and dimethylamine [126]. Cu vacancies in the catalyst promote CO_2_ adsorption, which spontaneously couples with DMA to form ^*^OCN^+^H(CH_3_)_2_O^−^ intermediates [126]. The introduction of Pd accelerates the electrochemical hydrogenation process, ultimately yielding DMF. Moreover, the N site can be progressively reduced to NH_2_ following the C─N coupling process, ultimately leading to the formation of an amide. TiO_2_‐supported Ru_1_Cu single‐atom alloy particles have demonstrated to electrochemically and selectively synthesize formamide from CO and NO_2_^−^ [123]. This process achieves remarkable formamide selectivity with a FE of 45.65 ± 0.76% at ‐0.5 V versus RHE. The adjacent Ru‐Cu dual active sites facilitate the spontaneous coupling of ^*^CO and ^*^NH_2_ intermediates, enabling the C─N coupling to produce ^*^CONOH (Figure 7b). This intermediate is subsequently reduced to form ^*^CONH and ^*^CONH_2_, ultimately yielding formamide. These studies provide innovative pathways for synthesizing high‐value amides using CO_2_ and waste water‐derived N sources.

Unlike electrochemical reduction for C─N coupling synthesis, electro oxidative C─N coupling often utilizes NH_3_ as the N source and alcohols as the C sources (Figure 7c). During the oxidation of alcohols, aldehyde‐like intermediates are generated, which are then nucleophilically attacked by NH_x_ species to form C─N bonds (Figure 7c). For instance, Pt‐based catalysts have been shown to electrochemically oxidize CH_3_OH and NH_3_ to produce formamide [127]. At a current density of 100 mA cm^−^^2^, the selectivity and FE for methanol‐to‐formamide conversion are 74.26% and 40.39%, respectively [127]. In this process, PtO_2_ on the Pt surface acts as the active component, facilitating the nucleophilic attack of NH_3_ on aldehyde‐like intermediates generated from methanol oxidation which followed by dehydration to form formamide through DFT calculation [127]. This strategy is also applicable to the synthesis of other organic nitrogen compounds, such as acetamide, acrylamide, and formyl methylamine. In contrast to Pt‐based catalysts, boron‐doped diamond (BDD) anodes have been used to synthesize HCONH_2_ via the electrooxidation of methanol and NH_3_, with H_2_C═NH as the key intermediate [128]. The reaction pathway involves the oxidation of methanol to an aldehyde intermediate, which reacts with NH_3_ to form an aldimine intermediate [128]. This intermediate is further oxidized to a nitrile, which is then hydrolyzed to formamide (Figure 7d). By scaling up the BDD electrode area to 2200 cm^2^, an FE of 33.5% and a formamide yield of 0.82 mol h^−^^1^ (36.9 g h^−^^1^) was achieved in an 8 L electrolysis device, demonstrating the potential for large‐scale green electrosynthesis of formamide [128]. Additionally, catalysts for more complex polyol systems have also been developed. For example, WO_3_ catalysts were used to convert alcohols, such as ethylene glycol (EG) from PET plastic waste and biomass‐derived polyols, into formamide in the presence of NH_3_ [129]. During the reaction, glycolaldehyde generated from EG oxidation is nucleophilically attacked by ^*^NH_2_, promoting C─C bond cleavage and C─N bond formation to yield formamide. The resulting formaldehyde is further oxidized to ^*^CHO, which reacts with ^*^NH_2_ to produce another formamide molecule (DFT calculation) [129]. This strategy was also extended to biomass‐derived polyols (e.g., glycerol and glucose), achieving FEs of 28%–46% and high yields of 60.0–231.8 µmol cm^−^^2^ h^−^^1^ [129]. Beyond NH_3_, organic N‐containing molecules can also participate in C─N coupling reactions to form amides. For example, a WO_2_‐NiOOH/Ni catalyst was developed for the co‐oxidation of methanol and DMA to produce DMF [130]. In this process, DMA is electro‐oxidized to (CH_3_)_2_N^*^ at WO_2_ active sites, while NiOOH promotes the oxidation of methanol to ^*^CHO, thus facilitating DMF synthesis [130].

In the typical electrosynthesis of amide, the formation of C─N bonds involves the nucleophilic attack of NH_x_ intermediates on aldehyde‐like intermediates. However, the complexity of amide compounds and the specific demands of industrial applications require further exploration of reaction mechanisms and the development of advanced electrosynthesis methods. In this context, pulse electrochemistry has emerged as a promising approach. Based on this technic, low‐coordinated Cu nano corals were used as the cathode to convert NO_2_^−^ and aryl boronic acids into aromatic amines [131]. The NO_2_^−^ was first reduced and hydrogenated to NH_3_. The anode potential was then switched to enable in situ Cu(II)‐catalyzed C─N coupling between NH_3_ and aryl boronic acids [131]. The pulse method maintains a high concentration of Cu(II) through in situ electrooxidation and significantly inhibits phenol byproduct formation by adjusting the solution pH [131]. This approach has also been applied to the efficient synthesis of ^15^N‐labeled aromatic amines, cycloaddition reactions, and click reactions, highlighting the broad application potential and market prospects of electrosynthesis technologies (Table 2).

**TABLE 2:** Heterogeneous catalysts used for electrosynthesis of amines, amino acids, and amides.

Catalyst	Reactants	Media	Product	Key C‐N intermediates	FE	Potential (V_RHE_)	Current density (*j*)	Yield	Stability duration (h)
CoPc‐NH_2_/CNT [25]	NO_3_^−^ +CO_2_	0.1 M KHCO_3_ + 0.5 M KNO_3_	Methylamine (CH_3_NH_2_)	NH_2_OH^*^ + HCHO^*^	13%	−0.92 V	35 mA/cm^2^	—	16
CoPc/CNT [100]	CO_2_ + Piperidine	0.1 M KHCO_3_+ 20 mM piperidine	N‐methylpiperidine	HCHO^*^+R‐NH	7.5%	−0.93 V	—	—	—
	CO_2_ + Methylamine	0.1 M KHCO_3_+ 20 mM Methylamine	Dimethylamine	HCHO^*^+H_3_C‐NH_2_	1%	−0.93 V	0.2 mA/cm^2^	—	12
	CO_2_ + Dimethylamine	0.1 M KHCO_3_+ 20 mM Dimethylamine	Trimethylamine	HCHO^*^+ (H_3_C)_2_‐NH	3.4%	−0.93 V	0.7 mA/cm^2^	—	—
	CO_2_ + NH_2_OH	0.1 M KHCO_3_+ 20 mM NH_2_OH	Methylamine	HCHO^*^+ NH_2_OH	9.8%	−0.93 V	2.5 mA/cm2	—	—
Oxide‐derived Cu NPs [101]	NO_3_^−^ + CO_2_	0.1 M KNO_3_ + 1.0 M KHCO_3_	Ethylamine (CH_3_CH_2_NH_2_)	NH_2_OH^*^ + CH_3_CHO^*^	0.3%	−1 V	70 mA/cm^2^	—	5
e‐OD‐Cu [102]	NO_3_^−^ + HCHO	0.1 M KOH + 0.5 M K_2_SO_4_ + 300 mM HCHO + 100 mM KNO_3_	Hexamethylenetetramine (HMTA)	NH_3_^*^ + HCHO^*^	74.9%	−0.3 V	∼85 mA/cm^2^	—	6 cycles
Cu‐Hg [108]	NO_3_^−^ + oxalic acid (OA)	15 wt% H_2_SO_4_ + 0.25 M OA and 0.25 M NaNO_3_	Glycine	NH_2_OH^*^ + COOHCHO^*^	43.1%	−1.4 V vs. (Ag/AgCl)	∼90 mA cm^−2^	—	2
Fe‐N‐C‐700 [109]	NO_3_^−^ + OA	0.5 M OA + 0.5 M NaNO_3_	Glycine	NH_2_OH^*^ + COOHCHO^*^	64.2%	−0.9 V	106.4 mA cm‐2	—	—
Fe/NC [111]	NO and α‐keto acids	0.1 M HCl + 20 mM 3‐methyl‐2‐oxobutanoic acid	Valine	NH_2_OH^*^ +α‐keto acids	—	−0.6 V	10 mA cm^−2^	32.1 µmol mg_cat_^−1^	19
CoFe‐SSM [112]	NO and α‐keto acids	0.1 M HCl + 4‐methyl‐2‐oxovaleric acid	Leucine	NH_2_OH^*^ +α‐keto acids	32.4%	−0.7 V	3.3317 g (2*2 cm^2^, 240 h, H‐cell)	115.4 µmol h^−1^	240
CoPc/CNT [113]	NO_3_^−^ and α‐keto acids	0.4 M H_2_SO_4_ + 0.7 M KNO_3_ + 0.2 M Pyr	Alanine	NH_2_OH^*^ +α‐keto acids	61%	−0.57 V	42 mA cm^−2^	—	1.11 (4000 s)
OD‐Ag [117]	NO + pyruvic acid (PA)	0.1 M NaOH+ 0.9 M NaClO_4_	Alanine	NH_2_OH^*^ + PA	70%	−0.56 V	100 mA cm^−2^	3.85 g (100 mA cm^−2^, 120 h, Flow cell)	120
F‐Cu_3_‐OF [116]	NO_3_^−^ + pyruvic acid	0.5 M KOH + 0.2 M NO_3_^−^ + 0.2 M pyruvic acid	Alanine	R‐COHCOO^*^ + ^*^NH	71%	−0.6 V	—	957 µmol cm^−2^ h^−1^	24
	NO_3_^−^ + 4‐methyl‐2‐oxovaleric acid	0.5 M KOH + 0.2 M NO_3_^−^ + 0.2 M 4‐methyl‐2‐oxovaleric acid	Leucine		57%	−0.6 V			
	NO_3_^−^ + 3‐methyl‐2‐oxobutanoic acid	0.5 M KOH + 0.2 M NO_3_^−^ + 0.2 M 3‐methyl‐2‐oxobutanoic acid	Valine		66%	−0.6 V			
	NO_3_^−^ + Phenylpyruvic acid	0.5 M KOH + 0.2 M NO_3_^−^ + 0.2 M phenylpyruvic acid	Phenylalanine		55%	−0.6 V			
	NO_3_^−^ + Glyoxalic acid	0.5 M KOH + 0.2 M NO_3_^−^ + 0.2 M glyoxalic acid	Glycine		42%	−0.6 V			
Cu NPs (GDL Load) [121]	NH_3_ + CO	1 M KOH	Acetamide	^*^C = C = O + NH_3_	40%	−0.68 V	300 mA cm^−2^	—	8
ER‐Cu [122]	HCOOH + NO_2_^−^	0.5 M NaOH + 0.2 M HCOOH +0.02 M NaNO_2_	Formamide	^*^CHO + ^*^NH_2_	29.7%	−0.4 V	—	35.1 mmol h^−1^ g_cat_^−1^	18
Ru_1_Cu SAA [123]	CO + NO_2_^−^	1 M KOH + 1 M KNO_2_	Formamide	^*^CO + ^*^NH_2_	45.65 ± 0.76%	−0.5 V	250 mA cm^−2^	2483.77 ± 155.34 µg h^−1^ mgcat‐1	50
CuO_x_/BiO_x_ [124]	CO_2_+NO_x_^−^	0.2 M KHCO_3_ + 0.02 M KNO_x_	Formamide (HCONH_2_)	^*^CHO + ^*^NH_2_	4.8 ± 0.4%	−3.0 V	125 mA cm^−2^	134 ± 11 mmol h^−1^ g_cat_^−1^	2
Cu NPs [125]	CO_2_ + NO_2_^−^	0.5 M KOH	Acetamide	NH_2_OH^*^ + CH_3_CHO	20%	−1.4 V	∼20 mA cm^−2^	—	—
Pd/Cu‐V_Cu_ [126]	CO + dimethylamine (DMA)	0.5 M KHCO_3_ + 1 M DMA	N, N‐Dimethylformamide (DMF)	*OCN^+^H(CH_3_)_2_O^−^	37.5%	—	100 mA cm^−2^	385 mmol·h^−1^·g_cat_^−1^	15
Pt‐Ti [127]	CH_3_OH + NH_3_	0.5 M NaHCO_3_ + CH_3_OH:NH_3_ = 4:1	Formamide (HCONH_2_)	CHO^*^ + NH_2_^*^	40.39%	—	100 mA cm^−2^	305.4 µmol·cm^−2^·h^−1^	46
BDD [128]	CH_3_OH + NH_3_	0.5 M NaHCO_3_ + CH_3_OH+ NH_3_	Formamide (HCONH_2_)	CHO^*^ + NH_2_^*^	41.2%	—	120 mA cm^−2^	461.39 µmol·cm^−2^·h^−1^	20 cycles
WO_3_ [129]	Ethylene glycol (EG) + NH_3_	0.25 M H_2_SO_4_ + EG+NH_3_	Formamide (HCONH_2_)	CHO^*^ + NH_2_^*^	43.2%	2.0 V	100 mA cm^−2^	537.7 µmol·cm^−2^·h^−1^ (flow cell)	12
WO_2_‐NiOOH [130]	CH_3_OH + DMA	0.5 M KHCO_3_ + CH_3_OH + DMA	N, N‐Dimethylformamide (DMF)	(CH_3_)_2_N^*^ + ^*^CHO	47%	—	100 mA cm^−2^	438 µmol·cm^−2^·h^−1^	80

## 3 Large‐Scale Production

**FIGURE 8:**
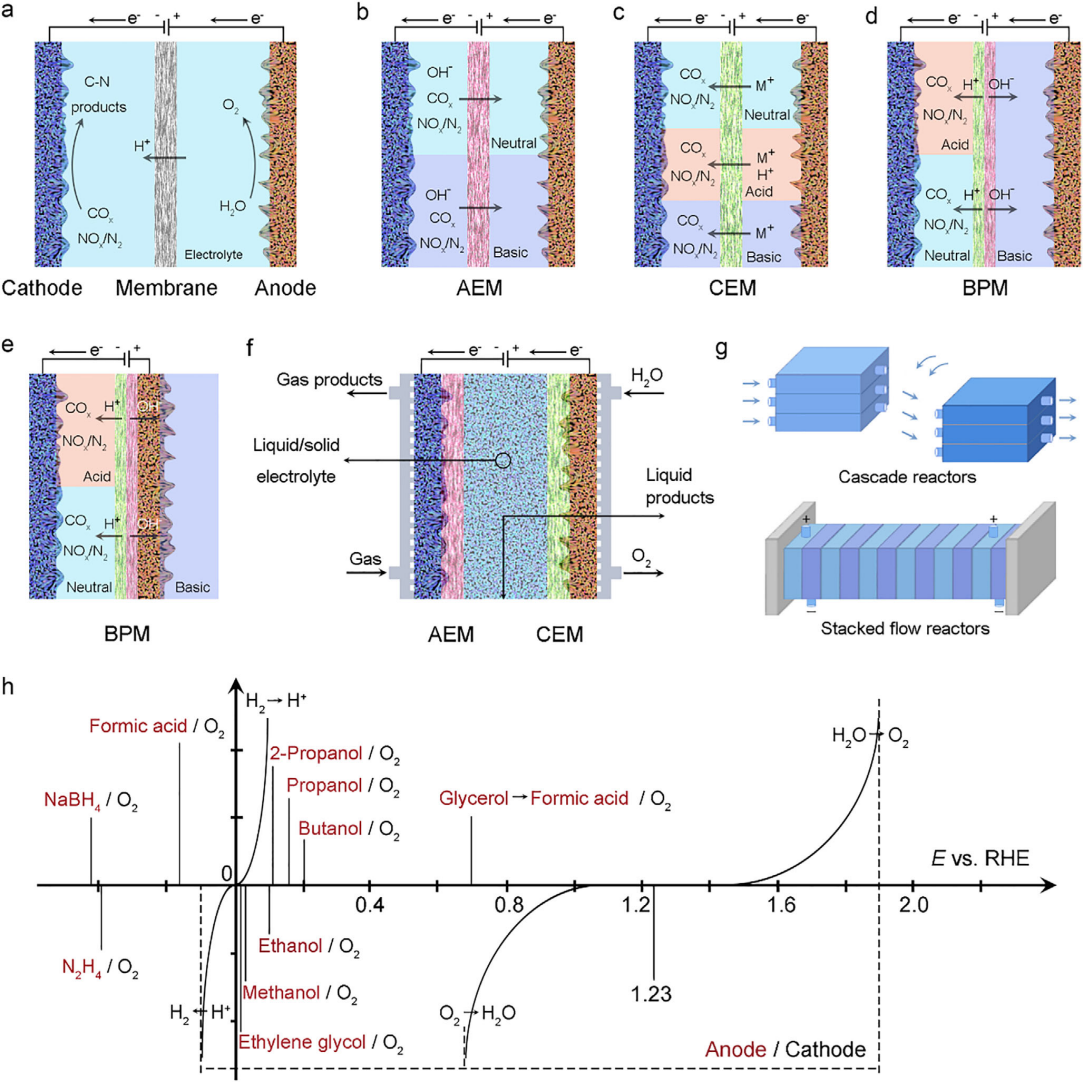
(a, b, c, d, e, f, g) Different Electrochemical cell configurations for C─N coupling reactions; (h) main categories of anode reactions that can replace the OER.

At this stage, rational design strategies for efficient C─N coupling reactions have been achieved through mechanistic insights, leading to enhanced intrinsic activity of electrocatalysts. However, beyond catalyst development, the evaluation device plays a critical role in determining catalytic performance. While various reactor configurations enable continuous synthesis of electrocatalytic products, conventional assessment methods often face limitations that restrict their practical applicability. Typically, electrocatalytic performance of C─N coupling is evaluated using a three‐electrode setup in an H‐type cell, where the basic structure of an electrolyzer includes a cathode, an anode, and a membrane positioned between them (Figure 8a). The C─N coupling reaction typically occurs on the catalyst surface, while the membrane isolates the electrodes and facilitates ion exchange [39, 68, 75, 132]. It is noted that the performance of H‐type cells is fundamentally limited by several critical factors: electrode surface hydration, restricted gas solubility, and reaction step mismatches. While H‐type cells are cost‐effective and operationally simple, they primarily serve as proof‐of‐concept tools for mechanistic studies. Their inherent design limitations prevent them from addressing the scalability requirements of industrial electrocatalysis. When gaseous reactants are involved, a GDE is often used, composed of a hydrophobic gas diffusion layer (GDL) and a hydrophilic catalyst layer. The GDL, a gas‐permeable support, allows reactants to reach the catalyst layer, where the reaction takes place. In liquid supply systems, reactants enter the electrolyzer in liquid form and hydrophilic electrodes are employed, making them suitable for C─N coupling reactions involving CO_x_ and NO_x_ or N_2_ [39, 132]. Due to the low solubility of reactive gases in aqueous electrolytes, these reactions typically operate at low current densities. Nevertheless, higher current densities can be achieved by increasing gas dissolution through optimized electrode design, pressurization, or in situ reactive gas generation on the electrode surface. By utilizing GDE, these systems establish a solid‐liquid‐gas triple‐phase interface, which enhances mass transfer, minimizes electrode surface hydration, and effectively suppresses the competing HER. Through extensive research, numerous advanced reaction cells have been developed based on H‐type electrolytic cells. Among these, electrochemical flow cells have emerged as a promising solution to overcome these challenges.

The choice of membrane, such as anion exchange membranes (AEMs), cation exchange membranes (CEMs), or bipolar membranes (BPMs), significantly impacts ion flow, reaction environment, and product crossover (Figure 8b–e). AEMs are commonly used in various electrocatalytic reactions, including water electrolysis and fuel cells. Compared with the classic H‐type electrolytic cell, the liquid flow cell assembled based on AEM can effectively improve the conversion rate of C─N coupling reaction. A comparative study between H‐type cells and flow cells focused on the electrosynthesis of urea via C─N coupling between N_2_ and CO_2_ revealed significant performance differences. In the traditional H‐cell setup, the maximum urea formation rate reached only 0.12 mmol g^−1^ h^−1^ with a FE of 0.66% at ‐0.4 V versus RHE [64]. In contrast, the flow cell system (AEM) delivered a dramatically improved urea yield of 3.36 mmol g^−1^ h^−1^ and an FE of 8.92% under identical conditions, highlighting its superior reaction kinetics and selectivity. For systems with soluble N sources, the AEM flow cell has also been shown to work effectively. The strategy employed Te‐doped Pd nanocatalysts for urea synthesis from CO_2_ and nitrite in an optimized flow cell achieved an impressive FE of ∼10.2% and an N‐efficiency of ∼82.3%, yielding a 0.95 wt% urea solution, demonstrating the critical role of flow cell configurations in mitigating mass transport limitations [80]. Additionally, an electrochemical method has been developed recently for synthesizing amino acids from nitric oxide and α‐keto acids [112]. While conventional H‐cells achieved only FE of 17% and yield of 11.45 mmol g^−1^ h^−1^ alanine due to mismatched reaction steps, similar reduction potentials, and poor mass transfer, these limitations were overcome by designing a spatially separated dual‐reactor system. Using an OD‐Ag catalyst, this system maintained >74% efficiency for pyruvate oxime production at 50–500 mA cm^−2^ and 70% alanine yield at 300 mA cm^−2^. Remarkably, under simulated sunlight, it produced 2.1 mmol pyruvate oxime and 1.7 mmol alanine over 8 h, demonstrating the promising synergy between electrochemical synthesis and renewable energy. However, AEM membranes may permit anionic products to cross, potentially leading to their oxidation back into reactants [39, 132, 133]. Similar as AEMs, CEMs are semipermeable barriers, but selectively permit the transport of positively charged ions (cations) while blocking anions and gases (Figure 8c). This selective ion transport helps maintain charge balance and regulate local reaction conditions, thereby stabilizing N‐intermediates during C─N coupling reactions. However, small molecules (e.g., NH_3_) may diffuse through membranes, thus reducing yield efficiency. BPMs demonstrate more comprehensive advantages, effectively compensating for the shortcomings of AEM and CEM, while also attracting growing scholarly attention. BPMs prevent membrane crossover and enable different pH environments (Figure 8d), though their high resistance can result in elevated cell voltages and reduced energy efficiency [39, 133]. Gas‐phase electrolyzers are categorized into gas‐phase systems and gas‐liquid cofeed systems. In gas‐phase systems, the GDE forms a zero‐gap electrolyzer with the membrane and anode, with all reactants in the gas phase. In gas‐liquid co‐feed systems, a liquid electrolyte containing co‐reactants is placed between the GDL and the membrane [133]. Wang and his colleagues developed a solid electrolyte reactor featuring three electrochemical chambers, which enables highly efficient nitrate reduction through a cation shielding effect [134]. Unlike conventional systems, this solid electrolyte electrocatalytic design eliminates the need for liquid electrolytes. The aqueous solution in the anode chamber permeates through the middle porous solid electrolyte layer, effectively blocking the electrochemical migration of alkali metal cations from the middle layer to the anode. This mechanism significantly enhances nitrate reduction selectivity, achieving a FE for ammonia synthesis of >90% while effectively suppressing competing HER. Due to these advantages, this improved BPM‐based electrolyzer demonstrates strong potential for large‐scale electrocatalytic C─N coupling applications.

Organic solvents, with their high gas solubility and unique reaction mechanisms, have emerged as promising electrolytes for C─N coupling [135] Liquid supply systems accommodate both polar and non‐polar organic solvents, while gas supply systems are limited to polar electrolytes (Figure 8f). When selecting organic solvents, factors such as ionic conductivity and membrane‐electrolyte compatibility must be considered. Although advancements in traditional alkaline electrolyzers and CO_2_ reduction systems provide valuable insights for C─N coupling, the efficiency of these reactions depends on multiple performance metrics, including catalytic selectivity, reaction activity, current density, energy efficiency, product concentration, product crossover, and stability [39, 131]. These factors collectively determine the economic viability and scientific value of C─N compounds. Consequently, the involvement of both gas and liquid reactants necessitates more complex electrolyzer configurations [133]. More sophisticated reactor designs, such as cascade reactors and stacked flow reactors, have been developed to address these challenges (Figure 8g). Cascade reactors allow reactants to fully interact at each stage, improving mass transfer efficiency [128, 131]. Each stage can be independently optimized, making cascade reactors suitable for complex reaction systems. By increasing the number of stages, these reactors can be scaled from small‐scale trials to industrial production. In contrast, stacked flow reactors feature a multi‐layer design that saves space and enables continuous feeding and discharging, making them ideal for large‐scale production [39, 128]. The gravity‐driven flow in stacked reactors reduces external power requirements and energy consumption. Additionally, their modular design simplifies maintenance and minimizes downtime. Beyond reactor design, reaction efficiency can be enhanced by optimizing anode reactions and reducing overpotentials [1, 136]. Common anode substitution reactions include the oxidation of organic small molecules such as methanol, ethanol, ethylene glycol, and formic acid (Figure 8h) [136]. The development of these anode reactions can significantly improve the intrinsic performance of C─N coupling systems. Whether through the design of new reactors or the development of anode replacement reactions, these advancements are poised to drive the large‐scale application of C─N coupled electrosynthesis systems in practical scenarios.

The optimization of electrolytic cell reactors for electrocatalytic C–N coupling focuses on enhancing reaction efficiency, improving product selectivity, and ensuring scalability. Achieving these goals requires a multi‐faceted approach integrating advances across molecular, reactor, and systems engineering scales. At the molecular level, precise design of catalysts and reaction pathways is foundational. Current strategies involve engineering specific active sites, such as single/dual metal sites or non‐metal‐doped carbon matrices and employing molecular mediation to regulate key intermediates and direct coupling selectivity. At the reactor engineering level, innovation in configuration and operation is critical for performance and scale‐up. Mass‐transfer‐limited reactions benefit significantly from flow cell architectures, while paired electrolysis strategies improve overall atom and energy economies by utilizing both half‐reactions. For practical translation, system‐level engineering and assessment are indispensable. The integration of techno‐economic analysis (TEA) and life cycle assessment (LCA) into the design process marks a pivotal shift in the field, moving beyond mere performance metrics toward sustainable and economically viable system designs. In conclusion, advancing electrocatalytic C–N coupling is a multi‐scale challenge.

## 4 Outlook and Perspective

Despite significant advancements in electrosynthesis which achieves a rapidly growing field enabling sustainable chemical production, its industrial adoption remains limited compared to conventional thermocatalytic methods. Key barriers include scalability challenges, energy efficiency constraints, and the complexity of competing reaction pathways. For high‐value C─N coupling products, critical hurdles persist in achieving sufficient selectivity, FE, and long‐term catalyst stability under operational conditions. Addressing these challenges requires innovative electrocatalyst design, optimized reactor engineering, and improved understanding of reaction mechanisms to bridge the lab‐to‐industry gap.

### 4.1 Improving Electrocatalytic Activity and Selectivity

The electrochemical co‐reduction of carbon sources (e.g., CO_2_, CO) and nitrogen sources (e.g., NO_3_^−^, NO_2_^−^), along with the co‐oxidation of alcohols and ammonia derivatives (NH_3_, NH_2_OH), has significantly broadened the possibilities for electrocatalytic synthesis of high‐value organic nitrogen compounds, such as amides, urea, and amines. However, the simultaneous activation and coupling of C and N species pose substantial challenges due to the need for precise temporal and spatial synchronization of reactive intermediates. This complexity often leads to competitive side reactions, including the undesired formation of single‐carbon (e.g., CH_4_, CO) or single‐nitrogen (e.g., N_2_, NH_3_) products, which reduces selectivity and Faradaic efficiency. To address these limitations, the rational design of electrocatalysts with multifunctional active sites is critical. Defect engineering including vacancies and edge sites precisely modulates electronic structures to stabilize C─N coupling intermediates, improving reaction efficiency. Heterogeneous interfaces (e.g., metal/oxide hybrids) create synergistic active sites, enabling simultaneous activation of C─ and N─containing reactants for enhanced catalytic performance. Additionally, atomic‐level catalysts, including single‐atom alloys and dual‐atom sites, offer unparalleled control over reaction pathways by fine‐tuning adsorption energetics for critical intermediates. Advanced characterization in situ spectroscopy techniques (e.g., in situ FTIR and Raman) and computational modeling further guide rational catalyst design, ensuring optimal active site configurations. By integrating defect engineering, interfacial synergy, and atomic precision, next‐generation catalysts can achieve superior activity, selectivity, and stability for sustainable C─N bond formation, advancing applications in energy storage and chemical synthesis.

### 4.2 Expansion of Catalytic Substrates

Broadening the substrate scope beyond conventional carbon and nitrogen sources is also imperative for advancing the industrial adoption of electrosynthesis. Electrocatalytic synthesis technology, driven by green electrical energy, demonstrates unique potential for industrialization in synthesizing organic nitrogen‐containing molecules from unconventional carbon/nitrogen sources (e.g., plastics, biomass waste). Its core advantages lie in the broad availability and economic viability of feedstocks and the green intensification of the process.

Electrocatalytic synthesis, driven by green electrical energy, holds unique potential for industrial‐scale production of organic nitrogen‐containing molecules from unconventional sources like plastic and biomass waste. Its advantages are rooted in economically viable, widely available feedstocks, and an inherently green, intensified process. Unlike traditional fossil‐dependent methods, this approach can directly utilize plastic pyrolysis products, biomass‐derived molecules, captured CO_2_, and industrial nitrogen waste. This shifts the feedstock base toward circular economy principles. The process is economically viable as it employs low‐cost, abundant waste streams, operates under mild conditions, reducing capital and energy costs. Notably, it uses modular reactors compatible with intermittent renewables and minimizes environmental costs by avoiding toxic reagents and excessive by‐products. Furthermore, precise control over electrode potential enables highly selective reactions with excellent atom economy. When implemented in continuous‐flow systems, it achieves intensified mass transfer and stable, high‐yield operation for industrial scale‐up.

### 4.3 Clarifying the Mechanism of Electrosynthetic C─N Coupling

Heterogeneous electrocatalytic reactions occur at the catalyst surface, where solid electrodes, liquid electrolytes, and gaseous reactants form dynamic two‐ or three‐phase interfaces. The complexity of these systems is amplified by the diversity of carbon‐ and nitrogen‐containing intermediates (e.g., *CO, *NH_2_, *CN) and competing C─N coupling pathways (e.g., nucleophilic attack, condensation), which often lead to unpredictable selectivity issues. To unravel these intricate mechanisms, advanced in situ/operando characterization techniques are essential for real‐time monitoring of intermediate adsorption, surface reconstruction, and reaction kinetics. These techniques enable the discovery of optimal reaction pathways and offer critical insights for the rational design of high‐performance catalysts. By systematically screening catalyst properties, materials can be identified to precisely meet reaction requirements, maximizing activity, and selectivity for desired C─N products. Advanced computational modeling and high‐throughput experimentation further accelerate catalyst optimization, ensuring efficient C─N coupling with minimal byproducts. Integrating mechanistic understanding with tailored material engineering allows for fine‐tuned adsorption and activation of intermediates, thus not only optimizing both activity and selectivity for target C─N products, but also guiding the development of sustainable and scalable synthesis routes.

### 4.4 Machine Learning and Artificial Intelligence Accelerates Catalyst Discovery

The rational design of tandem catalysts is crucial for electrocatalytic C─N coupling, as it directly addresses the core challenges of kinetic mismatch in the formation of carbon‐nitrogen intermediates and low coupling efficiency. By decoupling complex reactions into discrete, optimized steps at specific active sites, tandem catalysts can precisely control the local concentration and spatiotemporal proximity of key intermediates, thus overcoming the selectivity and activity limitations of single‐active‐site catalysts. Machine learning (ML) and artificial intelligence (AI) can perform high‐throughput virtual screening of catalyst composition and predict the adsorption energies of key intermediates [137]. Recent advances highlight the shift from descriptor‐based models to deep learning and large language models to explore a vast chemical space in catalysis [138].

Future catalytic system design will move beyond isolated component optimization to integrated membrane‐reactor‐electrode codesign. This may involve designing membranes with customized pore structures or surface chemistry to control local pH or reactant/product flux. Crucially, artificial intelligence can revolutionize the design process itself. For example, ML‐driven Bayesian optimization has been successfully applied to autonomously designing the geometry of flow reactors to enhance mixing and mass transfer, demonstrating a performance‐driven reactor creation paradigm [139]. The application of such codesign strategies is crucial for the scaling up of C─N coupled systems.

### 4.5 Designing Integrated Devices With High Efficiency and Low Energy Consumption

Beyond conventional approaches to device integration, emerging technologies, such as photo electrocatalysis and solid electrolyte electrolysis cells, provide valuable new paradigms. Photo electrocatalysis is unlocking innovative routes for the sustainable synthesis of organic nitrogen‐containing compounds. The synergistic use of light and electrical energy allows for the precise regulation of reactive intermediates under mild conditions, paving the way for advanced precision synthesis, including direct access to high‐value chiral amino acids. Solid electrolyte electrolysis cells integrate battery architecture with electro synthetic technology. By progressively aligning the target products of cathodic and anodic reactions, this configuration enhances overall product yield and device synthesis efficiency. All emerging engineering technologies have their final implementation manifested in practical device design and scalable production processes. The inherent complexity of C─N coupling systems necessitates innovative reactor designs that address multiple operational challenges simultaneously. The industrial translation of electrosynthesis extends beyond catalyst enhancement to the construction of a stable, efficient, and scalable integrated system. The long‐term stability of materials and components, especially separation membranes, must endure harsh industrial conditions, such as NO_x_ atmospheres, high potentials, and extreme pH. Under these operating conditions, the degradation, fouling, and swelling impair efficiency and lifespan. While electrode evolution, catalyst detachment, and support corrosion could further dictate reliability and cost. Besides, achieving system‐level energy efficiency and economic viability requires shifting from lab metrics like Faradaic efficiency to overall energy consumption per product, benchmarked against mature processes, like the Haber–Bosch method, through optimized stack design, minimized parasitic losses, integration of renewable electricity, and stability under dynamic operation with levelized cost ultimately determining industrial competitiveness.

Future reactors must achieve high single‐pass conversion rates while minimizing energy consumption through optimized mass/charge transport and overpotential reduction. Continuous flow systems with modular scalability show particular promise for industrial adoption, offering advantages in productivity and process control. Beyond conventional cascade and stacked flow reactors, emerging architectures incorporating advanced polymer membrane collectors enable precise intermediate confinement and pH gradient management. Hybrid designs integrating solid‐state electrolytes or GDEs further enhance performance by decoupling competing reactions. Such tailored engineering solutions will be critical for transitioning laboratory‐scale C─N coupling into economically viable production. Adopting proven continuous‐flow reactor architectures is essential to overcome mass‐transfer constraints, enable stable high‐current‐density operation, and facilitate scale‐up. Integrating these processes with renewable energy inputs and utilizing abundant feedstocks such as CO_2_, N_2_, or nitrogen oxides can establish fully integrated, low‐carbon manufacturing cycles, representing a critical path toward decarbonizing the chemical sector.

### 4.6 Analysis of Failure Mechanisms at Industrial Current Densities

While many electrocatalytic C─N coupling systems exhibit excellent selectivity at low current densities, the selectivity and stability typically decrease significantly, when the current density increases to industrially relevant levels (>200 mA cm^−2^). At high overpotentials, the kinetics of side reactions, such as the HER, are significantly enhanced, competing with the main C─N coupling reaction. At the same time, key intermediates with high surface coverage may be over‐hydrogenated, generating byproducts, leading to a decrease in the selectivity of the target product. To address this challenge, catalyst design must shift from simply pursuing high activity to precisely controlling the adsorption energy of intermediates. In high‐throughput electrolysis, the rate of reactant transport to the catalyst surface may not keep up with the electron transfer rate, leading to a decrease in reactant concentration on the electrode surface. More seriously, the rapid consumption of protons on the cathode surface can cause a sharp increase in the local pH value at the interface. This highly alkaline microenvironment can alter the reaction thermodynamics and may induce catalyst remodeling, ultimately resulting in a significant decrease in selectivity. Constructing a three‐phase reaction interface using a GDE is a key solution to the mass transfer problem of gaseous reactants. In addition, dynamic operation strategies such as pulse electrolysis can periodically refresh the reaction microenvironment on the electrode surface, which helps maintain stable selectivity at high current densities.

Efficient C─N coupling reactions require C─ and N─containing active intermediates to coexist on the catalyst surface with matched coverage. At high current densities, the difference in reduction kinetics between the two precursors can easily lead to excessive accumulation of one intermediate and insufficient supply of the other, thereby reducing coupling efficiency. The core strategy to solve this problem lies in designing active sites with spatial separation or functional synergy. For example, constructing dual‐site catalysts can preferentially activate CO_2_ and N_2_ species at different sites, and promote the migration and coupling of intermediates through proximity effects, thereby improving the reaction rate while maintaining high selectivity. Industrialization requires C─N coupling systems to simultaneously achieve high current density, high selectivity, and long‐term stability. Future research needs to deeply integrate mechanism exploration, reasonable catalyst design, and reactor engineering optimization. Only through such cross‐scale collaborative innovation can the selectivity bottleneck at high current densities be overcome, and electrosynthesis technology be promoted from the laboratory to practical applications.

### 4.7 Purification and Enrichment of Products

Electrocatalytic C─N coupling faces significant purification challenges as complex reaction networks typically yield mixed products (e.g., amines, amides, and nitriles), while downstream pharmaceutical and electronic applications demand high purity. Recent advances in tandem separation systems combining electrodialysis, chromatographic purification, and crystallization show promise for achieving pharmaceutical‐grade purity. Membrane‐based separation technologies with molecular‐level selectivity can simultaneously recover unreacted NO_x_ and CO_x_ feedstocks, improving atom economy. These integrated purification systems not only ensure product consistency, but also enable closed‐loop reagent recycling, reducing both energy consumption and waste generation. Such technological innovations are crucial for meeting industrial purity standards while maintaining cost competitiveness during scale‐up, ultimately bridging the gap between laboratory research and commercial production.

## Conflicts of Interest

The authors declare no conflicts of interest.

## Data Availability

Data sharing is not applicable to this article as no new data were created or analyzed in this study.
